# In situ Product Recovery of Microbially Synthesized Ethyl Acetate from the Exhaust Gas of a Bioreactor by Membrane Technology

**DOI:** 10.1002/elsc.202400041

**Published:** 2024-09-30

**Authors:** Andreas Hoffmann, Alexander Franz, Christian Löser, Thomas Hoyer, Marcus Weyd, Thomas Walther

**Affiliations:** ^1^ Chair of Bioprocess Engineering Institute of Natural Materials Technology Technische Universität Dresden Dresden Germany; ^2^ Interfaculty Centre for Bioactive Matter b‐ACT Matter Leipzig University Leipzig Germany; ^3^ Fraunhofer Institute for Ceramic Technologies and Systems IKTS Hermsdorf Germany

**Keywords:** delactosed whey permeate, ethyl acetate, in situ product recovery, *Kluyveromyces marxianus*, mixed matrix membrane, separation

## Abstract

Ethyl acetate is at present exclusively produced from fossil resources. Microbial synthesis of this ester from sugar‐rich waste as an alternative is an aerobic process. Ethyl acetate is highly volatile and therefore stripped with the exhaust gas from the bioreactor which enables in situ product recovery. Previous research on microbial formation of ethyl acetate has focused on the kinetics of ester synthesis and in part on the ester stripping, while the separation of the ester from the exhaust gas has hardly been investigated. A mixed matrix membrane was developed consisting of Silikalite‐1 embedded in polydimethylsiloxane which was installed in a radial–symmetrical membrane module. Evaluation of the separation of ethyl acetate was based on the analysis of the composition of the feed and retentate gas by mass spectrometry. The separation efficiency of the membrane was first tested with varied flows of artificial exhaust gas, containing defined amounts of ethyl acetate. A model for describing the separation process was parametrized by the measured data and used to design a real separation experiment. Ethyl acetate produced from delactosed whey permeate by *Kluyveromyces marxianus* DSM 5422 in a stirred bioreactor gassed with 0.5 vvm air was successfully separated from the exhaust gas by membranes; 93.6% of the stripped ester was separated. Liquid ethyl acetate was recovered by cooling the permeate gas to ‒78°C, whereby 99.75% of the condensed organic compounds were ethyl acetate. This study demonstrates for the first time that microbially produced and stripped ethyl acetate can be effectively separated from the exhaust gas of bioreactors by membrane technology to obtain the ester in high yield and purity.

**Abbreviations**
DWP: delactosed whey permeate
GC: gas chromatography
ISPR: in situ product recovery
MFC: mass flow controller
MMM: mixed matrix membrane
VOC: volatile organic compound

## 1 Introduction

Ethyl acetate is an important commodity chemical and is currently produced from fossil resources [1, 2]. In 2022, CropEnergies AG started the planning phase for the construction of a production plant for the synthesis of ethyl acetate from bioethanol via an ethanol dehydrogenation process [3]. Another alternative to petrochemical processes with economic potential is the microbial conversion of sugars directly to ethyl acetate [4]. The synthesis of ethyl acetate from sugar‐rich waste streams such as concentrated whey permeates [5, 6, 7] and even delactosed whey permeate (DWP) [8] has been successfully demonstrated using the Crabtree‐negative yeast *Kluyveromyces marxianus*.

**Summary**

- Ethyl acetate is currently produced exclusively from fossil resources.
- Microbial synthesis from sugar‐rich waste could be an interesting alternative. The produced ester evaporates due to its high volatility and is discharged from the bioreactor with the exhaust gas.
- This stripping enables an in situ product recovery, but the separation of ethyl acetate from the gas phase is a challenge.
- A membrane was developed that enables selective separation of the ester from the off‐gas. The membrane was characterized with regard to its separation performance under defined conditions using artificial exhaust gas.
- Finally, the membrane was used to separate ethyl acetate from a real bioreactor exhaust gas for the first time. 93.6% of the stripped ester was separated, yielding ethyl acetate of high purity.
- This membrane technology opens up a wide range of applications for the separation of volatile microbial products from the gas phase.

The microbial synthesis of ethyl acetate with *K. marxianus* is an aerobic process, during which the highly volatile ethyl acetate is rapidly transferred from the culture broth to the gas phase, leaving the bioreactor with the exhaust gas [5, 9]. This stripping counteracts the accumulation of ethyl acetate in the liquid phase to inhibitory concentrations [10]. In addition, the synthesis of ethyl acetate and its discharge from the cultivation medium occur simultaneously, allowing for in situ product recovery (ISPR). The concentration of ethyl acetate in the gas phase is determined by the partition coefficient and the concentration in the liquid phase, which typically does not exceed 5 g L^‒1^ [10] so that ethyl acetate reaches a maximum content of 0.025 L L^‒1^ in the gas.

Previous research on the microbial formation of ethyl acetate has focused on the kinetics of ester synthesis and on ester stripping, while the separation of the ester from the exhaust gas has rarely been investigated. Fredlund et al. [11] have separated ethyl acetate from the exhaust gas by absorption in decane, while Medeiros et al. [12] have separated this ester and other volatile organic compounds (VOCs) by adsorption on diverse materials; in both cases, only a small fraction of the formed ester has been separated, and the sorbed ester not [11] or only partially [12] recovered.

Several techniques could be used to separate ethyl acetate from gaseous mixtures. One approach is adsorption using materials such as activated carbon [12, 13, 14], polymeric resins [12], or molecular sieves [15]. However, the desorption process, which can involve temperature swing [16], pressure swing [17], steam [13], or solvent elution [12], is energy‐intensive and requires additional equipment. An alternative approach is a hybrid process in which ethyl acetate is separated from the gas by vapor permeation followed by condensation. Vapor permeation for the separation of VOCs from gas flows has been studied primarily for environmental purposes [18, 19, 20, 21]. The integration of vapor permeation for the recovery of fermentation products such as ethanol, butanol, and other lower alcohols is an emerging field with only a limited number of studies demonstrating its potential application [22, 23, 24].

The development of a process for the microbial production of ethyl acetate based on sugar‐rich residues also requires a technology for recovering the stripped ester. However, there have been no studies to date on the effective recovery of ethyl acetate from the exhaust gas of bioreactors. The present study demonstrates the ISPR of microbially synthesized ethyl acetate from the exhaust gas of a bioreactor by a mixed matrix membrane (MMM) for the first time. The recovery of the ester includes separation from the exhaust gas and subsequent condensation. The applied membrane was developed and produced for selective separation of ethyl acetate from the gas phase. Quantification of synthesis and separation of ethyl acetate is based on semi‐continuous measurement of the ester in gas flows by mass spectrometry. This quantification requires balancing of the ethyl acetate for the studied separation process. Then, a model is developed to describe the membrane separation process. Next, membrane separation is carried out by using artificial exhaust gas containing defined amounts of ethyl acetate to gain experience under defined conditions and to parameterize the model. Then, a real separation process is designed using the developed separation model. Finally, membrane separation of ethyl acetate from the exhaust gas and its subsequent condensation is demonstrated for a batch process in a stirred bioreactor with microbial production of ethyl acetate by *K. marxianus* based on DWP for the first time.

## 2 Materials and Methods

### 2.1 Membrane Preparation and Membrane Module Construction

The membrane and membrane module used in this work was designed and developed by the Fraunhofer Institute for Ceramic Technologies and Systems (IKTS Hermsdorf, Germany). The MMM was developed for the separation of ethyl acetate from the exhaust gas of bioreactors. The active separation layer of the MMM is composed of Silicalite‐1 particles and polydimethylsiloxane as the polymeric matrix. The zeolite Silicalite‐1 acts as an adsorbent for ethyl acetate. The membranes were prepared with a doctor blade coating system. The MMM layer was connected with the polyester fleece carrier by an intermediate layer of porous silicone rubber, yielding a mechanically stable membrane with a 10‐µm‐thick active layer (for details see Supporting Information 1).

The radial–symmetrical membrane module consists of four main components: (1) the circular membrane with a diameter of 154 mm, (2) a porous support plate made of sintered V4A steel on which the MMM rests, (3) an ethylene‐propylene‐diene monomer (EPDM) rubber seal, and (4) the outer module housing consisting of a lower and an upper part both made of polypropylene and connected to each other by six screws and wing nuts. The membrane exhibits an effective diameter of 148 mm, corresponding to a separation area of $A_{M}$ = 0.0172 m^2^. The exhaust gas is fed into the upper part of the housing via a central opening, the permeate gas is collected in a channel system below the support plate and leaves the module via a central opening in the lower part of the housing, and the retentate gas is collected in a ring‐shaped channel in the upper part of the housing and directed to the outside. The inner profile of the upper part of the housing is designed in such a manner that the flow velocity above the membrane is independent of the radius. For more details, refer to Supporting Information 1.

### 2.2 Evaluation of the Separation Process

The performance of the membrane to separate ethyl acetate from the other gas components of the exhaust gas was evaluated by the steady‐state flux of ethyl acetate through the membrane $J_{\mathrm{EA}}$, the separation yield $Y_{\mathrm{EA}}$, the separation factor $\alpha_{\mathrm{EA}}^{*}$, and the enrichment factor $E_{\mathrm{EA}}$. The area‐related flux through the membrane is calculated as follows (the superscript zero stands for standard conditions):

(1) $$J_{\mathrm{EA}}=\frac{F_{\mathrm{EA},M}^{0}}{A_{M}\cdot V_{m}^{0}}=\frac{x_{\mathrm{EA},\mathrm{perm}}\cdot F_{\mathrm{perm}}^{0}}{A_{M}\cdot V_{m}^{0}}$$

Herein, $F_{\mathrm{EA},M}^{0}$ is the flow of gaseous ethyl acetate through the membrane, $A_{M}$ is the surface area of the membrane, $V_{m}^{0}$ is the molar volume, and $x_{\mathrm{EA},\mathrm{perm}}$ is the volume fraction of ethyl acetate in the permeate gas. The separation yield is calculated as the ratio of the flow of ethyl acetate passing the membrane and the flow of ethyl acetate in the feed gas, $F_{\mathrm{EA},\mathrm{feed}}^{0}$. These two flows are each expressed as the total gas flow multiplied by the respective volume fraction of ethyl acetate

(2) $$Y_{\mathrm{EA}}=\frac{F_{\mathrm{EA},M}^{0}}{F_{\mathrm{EA},\mathrm{feed}}^{0}}=\frac{x_{\mathrm{EA},\mathrm{perm}}\cdot F_{\mathrm{perm}}^{0}}{x_{\mathrm{EA},\mathrm{feed}}\cdot F_{\mathrm{feed}}^{0}}$$

The separation factor of the membrane quantifies the degree to which ethyl acetate is concentrated in the permeate gas compared to its content in the feed gas. The separation factor is calculated as the ratio between the volume fraction of ethyl acetate and the volume fraction of all other components in the permeate divided by the same ratio for the feed gas [25]. The volume fraction of all other components besides ethyl acetate is expressed as the differences $\left(1-x_{\mathrm{EA},\mathrm{perm}}\right)$ and $\left(1-x_{\mathrm{EA},\mathrm{feed}}\right)$. This yields

(3) $$\alpha_{\mathrm{EA}}^{*}=\frac{x_{\mathrm{EA},\mathrm{perm}}\cdot \left(1-x_{\mathrm{EA},\mathrm{feed}}\right)}{x_{\mathrm{EA},\mathrm{feed}}\cdot \left(1-x_{\mathrm{EA},\mathrm{perm}}\right)}$$

The enrichment of ethyl acetate in the permeate gas in relation to the feed gas corresponds to the ratio of the ethyl acetate content in both gas flows, $x_{\mathrm{EA},\mathrm{perm}}$ and $x_{\mathrm{EA},\mathrm{feed}}$

(4) $$E_{\mathrm{EA}}=\frac{x_{\mathrm{EA},\mathrm{perm}}}{x_{\mathrm{EA},\mathrm{feed}}}$$

### 2.3 Separation of Ethyl Acetate from Artificial Exhaust Gas

**FIGURE 1:**
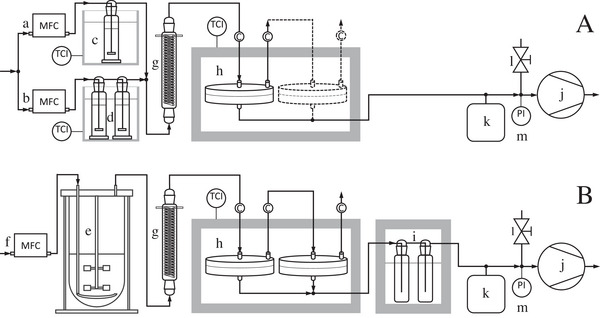
Experimental setup for separation of ethyl acetate (A) from artificial or (B) real exhaust gas; the artificial exhaust gas with a defined but variable ester content is produced by mixing two air flows both controlled by MFCs (a, b) and saturated with water at 25°C (c) or with ethyl acetate at 15°C (d); real exhaust gas originates from an aerated bioreactor (e) where the aeration flow is controlled by an MFC (f); the water content of the exhaust gases is restricted to 0.013 L L^‒1^ by condensers (g) operated at a dew point of water of 11°C; these exhaust gases are fed to membrane modules operated at 40°C in an insulation box (h), existing either as a single module or as two serial modules; the real permeate gas is passed through two cold traps operated at ‒78°C (i); the permeation of ethyl acetate through the membrane is driven by a pressure gradient maintained by a vacuum pump (j), whereby a buffer vessel (k) reduces pressure fluctuations and the vacuum is controlled by hand via a regulation valve (l) and pressure gauge (m); symbolizes sampling ports for measuring the content of ethyl acetate in gas flows by MS.

Artificial exhaust gas was generated in a setup shown in Figure 1A, consisting of two vapor generators and a condenser. One vapor generator produced air saturated with ethyl acetate, consisting of an E‐7100‐AAA mass flow controller (MFC) (Bronkhorst, The Netherlands, $F^{0}$ = 1.5 to 60 L h^‒1^) and two gas washing bottles with porosity 2 filter plates, each filled with 100 mL ethyl acetate (one bottle would have been sufficient for saturation, but the high volatility of ethyl acetate quickly reduces the quantity of ester). The other vapor generator produced air saturated with water, consisting of a GFC17 MFC (Aalborg, New York, USA, $F^{0}$ = 7.5 to 300 L h^‒1^) and one gas washing bottle with porosity 2 filter plate filled with 100 mL water. The gas‐washing bottles used had an H/D ratio of 4 and held a total volume of 175 mL. The supplied compressed air contained 0.0004 L L^‒1^ CO_2_. The MFCs had been calibrated using a 1000‐mL bubble flow meter (Supelco Analytical, Steinheim, Germany). The temperature of the gas‐washing bottles was controlled to 15°C (ethyl acetate) or 25°C (water) using two K10 cooling thermostats (Thermo Haake, Karlsruhe, Germany), resulting in an ester content of 0.0753 L L^‒1^ or water content of 0.0313 L L^‒1^ in the two generated gas flows. The two gas flows were mixed and resulted in a total flow with a specific content of ethyl acetate and water, which were adjusted via the setpoints of the MFCs. Then, the mixed flow passed through an intensive cooler (Lenz Laborglas, Wertheim, Germany) to cool the gas to 11°C for reducing the water content to 0.013 L L^‒1^ like the water content of real exhaust gas. Former experiments have shown that the retention of ethyl acetate by the condenser is negligible [5, 9]. The partially dehumidified gas was fed to the membrane module which was placed in an insulating box and its temperature was controlled to 40°C (in some experiments use of two modules which were connected to each other as shown in Figure 1). The ester was transported through the membrane due to a low pressure at the permeate side, which was generated by an MD4C vacuum pump (Vacuubrand, Wertheim, Germany, minimum $p_{\mathrm{perm}}$ = 5 mbar). The permeate pressure was regulated manually with a regulation valve and monitored with a DVR2 pro pressure gauge (Vacuubrand, Wertheim, Germany, reading accuracy 0.5 mbar). A buffer vessel (a desiccator with 2 L capacity) dampened pressure fluctuations and thus made it easier to set the permeate pressure on a desired value. PVC tubes were used for connecting the system components (wall thickness 2 mm, with fabric reinforcement in the vacuum area). The composition of the feed and retentate gas was measured by mass spectrometry while the low pressure of the permeate gas did not allow such quantification. Moreover, the retentate gas flow was measured with the bubble flow meter, and the permeate gas flow was determined by balancing and/or quantifying with the constant volume/variable pressure method (Supporting Information 2 [19, 26, 27, 28]).

### 2.4 Bioreactor Cultivation and Separation of Ethyl Acetate from Real Exhaust Gas

The yeast *K. marxianus* DSM 5422 was obtained from the Deutsche Sammlung von Mikroorganismen und Zellkulturen GmbH (Germany). Protocols for the preparation of the iron‐deficient DWP‐based medium and the inoculum are described in Hoffmann et al. [8]. For bioreactor cultivation, the same protocol as in [8] was followed with the only modification that the specific gassing rate was reduced from 1 to 0.5 vvm. The Labfors 5 bioreactor (Infors GmbH, Switzerland) with a total volume of 3.6 L was filled with 1 L medium and was gassed with 60 L h^‒1^ air given for standard conditions ($p^{0}$ = 1013.25 mbar and $T^{0}$ = 273.15 K).

The separation of ethyl acetate from the real exhaust gas by membrane technology is shown in Figure 1B. The exhaust gas is cooled to 11°C for partial dehumidification. The separation occurred with two membrane modules connected in series. The permeate flows of both modules were combined and then supplied to the condensation unit which consisted of two cold traps connected in series (capacity of 250 mL each; Rettberg GmbH, Göttingen, Germany) both cooled with dry ice in acetone to ‒78°C. The system for maintaining a pressure of 10 mbar on the permeate side was the same as used in the separation tests with artificial exhaust gas (see Section 2.3). The concentrations of ethyl acetate, ethanol, and acetaldehyde in the exhaust gas were measured by gas chromatography (GC), and the concentrations of ethyl acetate, ethanol, and water in the gas flow at the positions indicated in Figure 1B were measured by mass spectrometer (MS). The masses of products condensed in the cold traps were determined as follows: the condensate was dissolved in water, the solution filled up with water to a defined volume and analyzed by GC (mass of product = concentration of product × volume of filled up solution).

### 2.5 Analytical Methods

Protocols for quantification of biomass dry weight, dissolved non‐volatile compounds (lactose, galactose, glycerol, lactate, citrate, and acetate) by HPLC and VOCs (ethyl acetate, ethanol, and acetaldehyde) in the culture medium and in the exhaust gas by GC are described in Hoffmann et al. [8].

Gas phase concentrations, especially in the gas flows during membrane separation, were also quantified in a quasi‐continuous mode by mass spectrometry (GAM 2000, InProcess Instruments, Germany). Gas samples were supplied to the MS through a specific sampling station directly connected to the feed‐gas and retentate‐gas line of the membrane modules. The gas sample was analyzed by a secondary electron multiplier detector. The following m/z ratios were evaluated: 18 for water, 28 for N_2_, 32 for O_2_, 40 for Ar, 28 and 44 for CO_2_, 28 and 31 for ethanol, and 28, 31, 44 and 88 for ethyl acetate. The specific construction of the MS, the applied analysis protocol, and the calibration are detailed in Supporting Information 2.

## 3 Results and Discussion

Studies on the whey‐based production of ethyl acetate have so far focused on the microbial process, with emphasis on microbial strains and optimization of the cultivation conditions (for an overview see [8, 29]). This work concentrates on the downstream process, specifically exploring a method for the ISPR of ethyl acetate. This recovery method combines stripping, membrane separation, and condensation techniques. The stripping of ethyl acetate has been studied in detail previously [5, 9]. This work focuses specifically on evaluating and optimizing the separation performance of a developed membrane.

### 3.1 Balancing of the Membrane Separation Process

Balancing the separation of a specific component from a gas mixture by a membrane is usually based on measuring the concentration of the relevant component in the feed and in the permeate gas. Various measuring methods such as direct GC analysis [30, 31, 32] or constant volume/variable pressure methods [19, 26, 27, 28] have been used. These methods rely on the use of cold traps for quantification and are thus discontinuous. Since the ethyl acetate content in the exhaust gas of the bioreactor changes rapidly in time, a method for quasi‐continuous monitoring of the membrane performance is most appropriate. However, the quasi‐continuous measurement of ethyl acetate in the permeate gas is complicated due to the low permeate pressure. A balancing method based on semi‐continuous analysis of ethyl acetate in the feed and retentate was therefore developed as an alternative.

The membrane separation process involves three gas flows: the feed ($F_{\mathrm{feed}}^{0}$), the permeate ($F_{\mathrm{perm}}^{0}$), and the retentate ($F_{\mathrm{ret}}^{0}$). The feed flow is equal to the sum of the retentate and permeate flows when considering the flows at standard conditions (at $p^{0}$ = 1013.25 mbar and $T^{0}$ = 273.15 K): $F_{\mathrm{feed}}^{0}=F_{\mathrm{perm}}^{0}+F_{\mathrm{ret}}^{0}$. Such balancing is also possible for a single compound of a gas mixture as, for example, for ethyl acetate:

(5) $$F_{\mathrm{EA},\mathrm{feed}}^{0}=F_{\mathrm{feed}}^{0}\cdot x_{\mathrm{EA},\mathrm{feed}}=F_{\mathrm{perm}}^{0}\cdot x_{\mathrm{EA},\mathrm{perm}}+F_{\mathrm{ret}}^{0}\cdot x_{\mathrm{EA},\mathrm{ret}}$$

Herein, $x_{\mathrm{EA},j}$ is the volume fraction (equal to the molar fraction) of ethyl acetate in gas flow j. $x_{\mathrm{EA},\mathrm{feed}}$ and $x_{\mathrm{EA},\mathrm{ret}}$ were measured by MS, but $x_{\mathrm{EA},\mathrm{perm}}$ was not measurable. Substitution of $F_{\mathrm{ret}}^{0}$ by the difference $F_{\mathrm{feed}}^{0}-F_{\mathrm{perm}}^{0}$ in Equation (5) and rearrangement yields:

(6) $$x_{\mathrm{EA},\mathrm{perm}}=\frac{F_{\mathrm{feed}}^{0}\cdot x_{\mathrm{EA},\mathrm{feed}}-\left(F_{\mathrm{feed}}^{0}-F_{\mathrm{perm}}^{0}\right)\cdot x_{\mathrm{EA},\mathrm{ret}}}{F_{\mathrm{perm}}^{0}\;}$$

Equation (6) is also valid for other compounds when index EA is substituted by another component. Water passes through the membrane like ethyl acetate so that $x_{H_{2}O,\mathrm{perm}}$ is also of interest. The permeate flow, $F_{\mathrm{perm}}^{0}$, in Equation (6) is also unknown and must be determined. This flow is composed of the flows of three major compounds through the membrane: inert gas ($F_{\mathrm{inert},M}^{0}$), water vapor ($F_{H_{2}O,M}^{0}$) and ethyl acetate ($F_{\mathrm{EA},M}^{0}$), yielding:

(7) $$F_{\mathrm{perm}}^{0}=F_{\mathrm{inert},M}^{0}+F_{H_{2}O,M}^{0}+F_{\mathrm{EA},M}^{0}$$

The inert gas consists of nitrogen, oxygen, and carbon dioxide as the constituents of the exhaust gas from bioreactors. Substitution of $F_{\mathrm{EA},M}^{0}$ and $F_{H_{2}O,M}^{0}$ in Equation (7) with $F_{\mathrm{perm}}^{0}\cdot x_{\mathrm{EA},\mathrm{perm}}$ and $F_{\mathrm{perm}}^{0}\cdot x_{H_{2}O,\mathrm{perm}}$, and further substitution of $x_{\mathrm{EA},\mathrm{perm}}$ and $x_{H_{2}O,\mathrm{perm}}$ using Equation (6) results in:

(8) $$F_{\mathrm{perm}}^{0}=\frac{F_{\mathrm{feed}}^{0}\cdot \left(x_{\mathrm{EA},\mathrm{feed}}-x_{\mathrm{EA},\mathrm{ret}}+x_{H_{2}O,\mathrm{feed}}-x_{H_{2}O,\mathrm{ret}}\right)+F_{\mathrm{inert},M}^{0}}{1-x_{\mathrm{EA},\mathrm{ret}}-x_{H_{2}O,\mathrm{ret}}}$$

The inert gas flow $F_{\mathrm{inert},M}^{0}$ depends on the membrane characteristics and the pressure gradient over the membrane $p_{\mathrm{feed}}-p_{\mathrm{perm}}$. $F_{\mathrm{inert},M}^{0}$ was measured for a varied pressure gradient using the constant volume/variable pressure method with air, nitrogen, or carbon dioxide as the test gases. $F_{\mathrm{inert},M}^{0}$ was neither influenced by the type of the test gas nor affected by the presence of ethyl acetate in the feed gas (Supporting Information 3). In detail, the measurements demonstrated that $F_{\mathrm{inert},M}^{0}$ was not changed by $x_{\mathrm{EA},\mathrm{feed}}$ values from 0 to 0.045 L L^‒1^. Yang et al. [19] also found that $F_{\mathrm{inert},M}^{0}$ is not influenced by ethyl acetate at $x_{\mathrm{EA},\mathrm{feed}}$ values of up to 0.1 L L^‒1^.

Equations (6) and (8) enable the determination of $F_{\mathrm{perm}}^{0}$ and $x_{\mathrm{EA},\mathrm{perm}}$ based on known ($F_{\mathrm{feed}}^{0}$), predetermined ($F_{\mathrm{inert},M}^{0}$ as a function of $p_{\mathrm{perm}}$) and measured ($x_{\mathrm{EA},\mathrm{feed}}$, $x_{\mathrm{EA},\mathrm{ret}}$, $x_{H_{2}O,\mathrm{feed}}$, $x_{H_{2}O,\mathrm{ret}}$) variables.

### 3.2 Modeling of the Membrane Separation Process

The modeling of membrane separation served several purposes: (a) interpretation of the results from separation experiments with artificial exhaust gas (therefore parameterization of the model largely independent of these separation tests), (b) supporting the design of the real separation process, (c) comparison of the results from the real separation test with the model predictions, and (d) future optimization and scale‐up of separation processes. Previously published models are based on rectangular membranes with lateral feed and cross‐flowing permeate gas (e.g., in [20, 21, 33]). Here, a radially symmetrical membrane with central gas supply was used, and a sintered plate beneath the membrane prevented cross‐flow of the permeate gas. The flow regime on the permeate side significantly influences the gradient across the membrane.

#### 3.2.1 Model Development

This modeling was aimed at interpreting the measured separation data with artificial exhaust gas and was used for designing the real separation process. The transport of ethyl acetate through a membrane can be described by three consecutive steps: (1) adsorption on the upstream side of the membrane, (2) diffusive transport through the membrane material, and (3) desorption from the downstream side of the membrane [34, 35]. The membrane module is radially symmetrical and ensures radial transport of the feed gas from the center to the outer edge of the membrane (Supporting Information 1). All parameters therefore depend on radius r. The transport of ethyl acetate through the membrane is diffusive, so that flux $J_{\mathrm{EA}}\left(r\right)$ is described by Fick's first law:

(9) $$J_{\mathrm{EA}}\left(r\right)=D_{\mathrm{EA},M}\cdot \frac{C_{\mathrm{EA},M,\mathrm{up}}\left(r\right)-C_{\mathrm{EA},M,\mathrm{down}}\left(r\right)}{z_{M}}$$

Herein, $D_{\mathrm{EA},M}$ is the diffusion coefficient, $z_{M}$ is the thickness of the membrane, and $C_{\mathrm{EA},M,\mathrm{up}}\left(r\right)$ and $C_{\mathrm{EA},M,\mathrm{down}}\left(r\right)$ are the concentrations of the ester on the upstream interface and downstream interface at radius r. For modeling the sorption and desorption step, the Henry sorption model was used since this model described the studied sorption process best and generally provides a reasonable approximation at low concentrations [36]. The Henry solubility constant usually quantifies the linear relation between the liquid‐phase concentration and the partial pressure in the gas phase of the considered compound at the phase boundary. Here, the Henry coefficient $H_{\mathrm{EA},M/G}$ characterizes the relation between the concentration of ethyl acetate in the membrane material and the partial pressure of ethyl acetate in the gas at the interface. This relationship can be expressed for the upstream and downstream side: $C_{\mathrm{EA},M,\mathrm{up}}\left(r\right)=H_{\mathrm{EA},M/G}\cdot p_{\mathrm{EA},\mathrm{up}}\left(r\right)$ and $C_{\mathrm{EA},M,\mathrm{down}}\left(r\right)=H_{\mathrm{EA},M/G}\cdot p_{\mathrm{EA},\mathrm{down}}\left(r\right)$. The partial pressures of ethyl acetate in the gases are $p_{\mathrm{EA},\mathrm{up}}\left(r\right)=x_{\mathrm{EA},\mathrm{up}}\left(r\right)\cdot p_{\mathrm{feed}}$ and $p_{\mathrm{EA},\mathrm{down}}\left(r\right)=x_{\mathrm{EA},\mathrm{down}}\left(r\right)\cdot p_{\mathrm{perm}}$. Combination of these four relations with Equation (9) and substitution of term $D_{\mathrm{EA},M}\cdot H_{\mathrm{EA},M/G}$ by the permeability $P_{\mathrm{EA},M}$ [25] gives:

(10) $$J_{\mathrm{EA}}\left(r\right)=\frac{P_{\mathrm{EA},M}}{z_{M}}\cdot \left(x_{\mathrm{EA},\mathrm{up}}\left(r\right)\cdot p_{\mathrm{feed}}-x_{\mathrm{EA},\mathrm{down}}\left(r\right)\cdot p_{\mathrm{perm}}\right)$$

Herein, $x_{\mathrm{EA},\mathrm{up}}\left(r\right)$ and $x_{\mathrm{EA},\mathrm{down}}\left(r\right)$ are the volume fractions of ethyl acetate in the gases on the upstream and downstream side at radius r of the circular membrane, $p_{\mathrm{feed}}$ is the feed pressure (nearly identical with the ambient pressure), and $p_{\mathrm{perm}}$ is the permeate pressure.

Due to the rotationally symmetrical design of the membrane, the process variables change over the radius r: The feed gas enters the module at the center and flows radially across the membrane, whereby ethyl acetate is partially adsorbed by the membrane material. In this process, the volume fraction of ethyl acetate, $x_{\mathrm{EA},\mathrm{up}}$, as well as the gas flow, $F_{\mathrm{up}}^{0}$, change with the radius:

(11) $$\frac{dx_{\mathrm{EA},\mathrm{up}}}{dr}=-\frac{J_{\mathrm{EA}}\left(r\right)}{F_{\mathrm{up}}^{0}\left(r\right)}\cdot V_{m}^{0}\cdot 2\cdot \pi \cdot r$$

(12) $$\frac{dF_{\mathrm{up}}^{0}}{dr}=-\left(J_{\mathrm{inert}}+J_{\mathrm{EA}}\left(r\right)\right)\cdot V_{m}^{0}\cdot 2\cdot \pi \cdot r$$

Besides inert gas and ethyl acetate, some water passes through the membrane, but this flux is neglected in Equation (12) for reasons of simplification. The volume fraction of ethyl acetate on the downstream side is calculated as follows:

(13) $$x_{\mathrm{EA},\mathrm{down}}\left(r\right)=\frac{J_{\mathrm{EA}}\left(r\right)}{J_{\mathrm{inert}}+J_{\mathrm{EA}}\left(r\right)}$$

The inert gas passing through the membrane dilutes the transported ethyl acetate on the permeate side and thus reduces $x_{\mathrm{EA},\mathrm{down}}\left(r\right)$. $J_{\mathrm{inert}}$ is not a function of the radius. The total permeate gas flow, $F_{\mathrm{perm}}^{0}$, is obtained by integrating the differential gas flow $dF_{\mathrm{down}}^{0}\left(r\right)$ over the membrane area. And $dF_{\mathrm{down}}^{0}\left(r\right)$ corresponds to the decrease of the gas flow on the upstream side: $dF_{\mathrm{up}}^{0}\left(r\right)=-dF_{\mathrm{down}}^{0}\left(r\right)$. Using Equation (12) yields:

(14) $$F_{\mathrm{perm}}^{0}=\int_{r=0}^{r=r_{\max}}\left(J_{\mathrm{inert}}+J_{\mathrm{EA}}\left(r\right)\right)\cdot V_{m}^{0}\cdot 2\cdot \pi \cdot r\;dr$$

The differential gas flows $dF_{\mathrm{down}}^{0}\left(r\right)$ with individual values of $x_{\mathrm{EA},\mathrm{down}}\left(r\right)$ according to Equation (13) are mixed at the permeate outlet of the membrane module resulting in the gas flow $F_{\mathrm{perm}}^{0}$ having an average content of ethyl acetate, $x_{\mathrm{EA},\mathrm{perm}}$. The value of $x_{\mathrm{EA},\mathrm{perm}}$ is therefore calculated as the weighted average of $x_{\mathrm{EA},\mathrm{down}}\left(r\right)$ of all differential gas flows $dF_{\mathrm{down}}^{0}\left(r\right)$:

(15) $$x_{\mathrm{EA},\mathrm{perm}}=\frac{\int_{r=0}^{r=r_{\max}}x_{\mathrm{EA},\mathrm{down}}\left(r\right)\;dF_{\mathrm{down}}^{0}}{\int_{r=0}^{r=r_{\max}}dF_{\mathrm{down}}^{0}}$$

The integral $\int_{r=0}^{r=r_{\max}}dF_{\mathrm{down}}^{0}$ is identical with $F_{\mathrm{down}}^{0}$ which equates to $F_{\mathrm{perm}}^{0}$ calculable with Equation (14). Moreover, $dF_{\mathrm{down}}^{0}$ corresponds to $-dF_{\mathrm{up}}^{0}.$ Substitution of $dF_{\mathrm{up}}^{0}$ with Equation (12) and substitution of $x_{\mathrm{EA},\mathrm{down}}\left(r\right)$ with Equation (13) in the numerator of Equation (15) and subsequent transformation of the resulting expression gives:

(16) $$x_{\mathrm{EA},\mathrm{perm}}=\frac{2\cdot \pi \cdot V_{m}^{0}}{F_{\mathrm{perm}}^{0}}\cdot \int_{r=0}^{r=r_{\max}}J_{\mathrm{EA}}\left(r\right)\cdot r\;dr$$

#### 3.2.2 Process Simulation

The derived system of four differential equations (Equations (10), (11), (12), (13)) and two integrals (Equations 14 and 16) has to be solved simultaneously for calculating the six wanted process variables $J_{\mathrm{EA}}\left(r\right)$, $x_{\mathrm{EA},\mathrm{up}}\left(r\right)$, $x_{\mathrm{EA},\mathrm{down}}\left(r\right)$, $F_{\mathrm{up}}^{0}\left(r\right)$, $F_{\mathrm{perm}}^{0}$, and $x_{\mathrm{EA},\mathrm{perm}}$. The number of these process variables corresponds to the number of independent equations as expected. The model contains the following parameters: $z_{M}=10^{-5}\;m$, $r_{\max}=0.074\;m$ (constructive parameters), $p_{\mathrm{feed}}$, $p_{\mathrm{perm}}$, and $J_{\mathrm{inert}}$ (depending on the adjusted process conditions), $V_{m}^{0}$ = 22.414 L mol^‒1^ (a constant), and $P_{\mathrm{EA},M}$ (an intrinsic material property of the membrane).

The area‐specific inert gas flux, $J_{\mathrm{inert}}$, is calculated from the absolute inert gas flow, $F_{\mathrm{inert},M}^{0}$:

(17) $$J_{\mathrm{inert}}=\frac{F_{\mathrm{inert},M}^{0}}{A_{M}\cdot V_{m}^{0}}$$

In membranes without macroscopic membrane defects, the inert gas passes through microscopic defects in the polymeric membrane material [37]. The dependence of $F_{\mathrm{inert},M}^{0}$ on the pressure gradient ($p_{\mathrm{feed}}-p_{\mathrm{perm}}$) is then described as follows (see Supporting Information 3):

(18) $$F_{\mathrm{inert},M}^{0}=\frac{p_{\mathrm{feed}}\cdot T^{0}}{p^{0}\cdot T_{M}}\cdot A_{M}\cdot {\left(\frac{2\cdot \left(p_{\mathrm{feed}}-p_{\mathrm{perm}}\right)}{\rho_{\mathrm{feed}}\cdot \zeta_{M}^{*}}\right)}^{0.5}$$

Herein, $\rho_{\mathrm{feed}}$ is the density of the supplied gas at process conditions and $\zeta_{M}^{*}$ is the pressure loss coefficient of the membrane (for their determination see Supporting Information 3).

The simulation of the process was done by simultaneously solving Equations (10) to (13), (14) and (16) using the ordinary differential equation solver ode15s of Matlab (Mathworks, Natick MA, USA). The following boundary conditions were applied:$\;x_{\mathrm{EA},\mathrm{up}}\left(r\;=0\right)=x_{\mathrm{EA},\mathrm{feed}}$ and $F_{\mathrm{up}}^{0}\left(r\;=0\right)=F_{\mathrm{feed}}^{0}$. $J_{\mathrm{EA}}\left(r\;=0\right),$ and $x_{\mathrm{EA},\mathrm{down}}\left(r\;=0\right)$ were determined by solving Equations (10) and (13) iteratively for r=0 using the decic function of Matlab.

### 3.3 Separation of Ethyl Acetate from Artificial Exhaust Gas

The separation performance of the membrane was tested using artificial exhaust gas prior to its application in a biotechnological process. Separation of ethyl acetate from artificial exhaust gas exhibits several advantages: the flow as well as the ethyl acetate content of the feed gas can be set on desired specified values and both parameters remain constant during the test (measurement under steady‐state conditions).

#### 3.3.1 Preliminary Considerations

The exhaust‐gas generator shown in Figure 1A enabled the setting $F_{\mathrm{feed}}^{0}$ and $x_{\mathrm{EA},\mathrm{feed}}$ on specified values by variation of the set points of the two MFCs a and b. The actual values of $F_{\mathrm{feed}}^{0}$ and $x_{\mathrm{EA},\mathrm{feed}}$ were measured using the bubble flow meter and MS, respectively. The water content of the gas always amounted to 0.013 L L^‒1^, like the water content of the real exhaust gas.

First, the dynamic behavior of the exhaust‐gas generator was tested at a sudden change of the set points of in the MFCs. Steady‐state values for $F_{\mathrm{feed}}^{0}$ were obtained immediately, while the time required to reach constant $x_{\mathrm{EA},\mathrm{feed}}$ values depended on the gas flow, but never lasted longer than 15 min.

The evaluation of the separation process was based on four parameters defined by Equations (1) to (4) which were calculated from known data ($A_{M}$, $V_{m}^{0}$, $F_{\mathrm{feed}}^{0}$, $x_{\mathrm{EA},\mathrm{feed}}$) and the variables $F_{\mathrm{perm}}^{0}$ and $x_{\mathrm{EA},\mathrm{perm}}$ which characterize the permeate gas. The values of $F_{\mathrm{perm}}^{0}$ and $x_{\mathrm{EA},\mathrm{perm}}$ could not be measured due to the low $p_{\mathrm{perm}}$ but were calculated from known data ($F_{\mathrm{feed}}^{0}$, $x_{\mathrm{EA},\mathrm{feed}}$, $x_{H_{2}O,\mathrm{feed}}$), measured MS data ($x_{\mathrm{EA},\mathrm{ret}}$, $x_{H_{2}O,\mathrm{ret}}$), and the inert gas flow passing the membrane ($F_{\mathrm{inert},M}^{0}$) using Equations (6) and (8).

This method of $F_{\mathrm{perm}}^{0}$ determination was checked for reliability by comparing calculated $F_{\mathrm{perm}}^{0}$ values with measured $F_{\mathrm{perm}}^{0}$ data. $F_{\mathrm{perm}}^{0}$ was measured for various $F_{\mathrm{feed}}^{0}$ and $x_{\mathrm{EA},\mathrm{feed}}$ values using the constant volume/variable pressure method, or was determined indirectly as $F_{\mathrm{perm}}^{0}=F_{\mathrm{feed}}^{0}-F_{\mathrm{ret}}^{0}$, whereby $F_{\mathrm{ret}}^{0}$ was measured with the bubble flow meter. The calculated $F_{\mathrm{perm}}^{0}$ data matched well the measured flows (Supporting Information 3).

**FIGURE 2:**
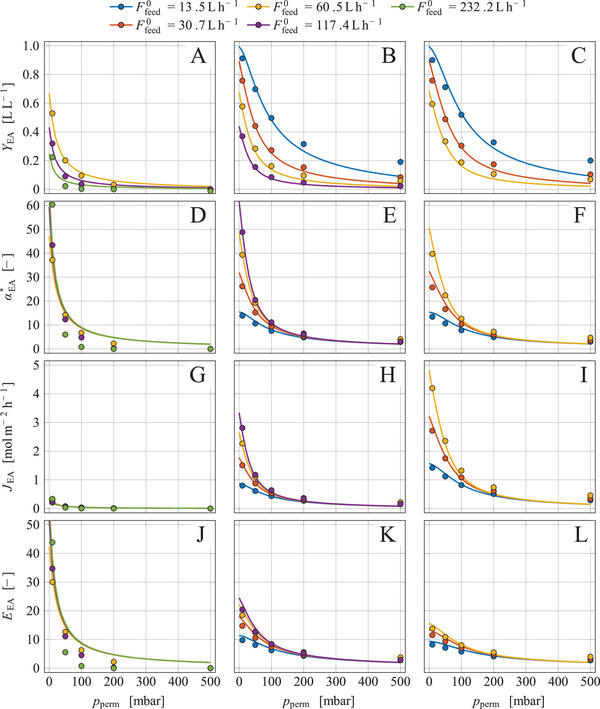
Membrane performance parameters $J_{\mathrm{EA}}$, $Y_{\mathrm{EA}}$, $\alpha_{\mathrm{EA}}^{*}$, and $E_{\mathrm{EA}}$ of a single membrane module at various permeate pressures and feed gas flows using artificial exhaust gas; the artificial feed gas based on air contained a varying amount of ethyl acetate, namely (A, D, G, J) $x_{\mathrm{EA},\mathrm{feed}}$ = 0.0025 L L^‒1^, (B, E, H, K) $x_{\mathrm{EA},\mathrm{feed}}$ = 0.025 L L^‒1^, or (C, F, I, L) $x_{\mathrm{EA},\mathrm{feed}}$ = 0.045 L L^‒1^; the feed‐gas flow $F_{\mathrm{feed}}^{0}$ was varied as indicated in the legend; symbols mark data based on measurements while solid lines represent model simulations; conditions and parameters: $x_{H_{2}O,\mathrm{feed}}$ = 0.013 L L^−1^, $T_{M}$ = 40°C, $p_{\mathrm{feed}}$ = 998.5 mbar, $P_{\mathrm{EA},M}$ = 4.7·10^‒7^ mol s^‒1^ m^‒1^ bar^‒1^, $A_{M}$ = 0.0172 m^2^, $\zeta_{M}^{*}$ = 6.0·10^14^.

The inert gas flow $F_{\mathrm{inert},M}^{0}$ required for $F_{\mathrm{perm}}^{0}$ determination was calculated using Equation (18) for conditions that prevailed during the separation experiments (see caption in Figure 2). At permeate pressures of $p_{\mathrm{perm}}$ = 10 to 500 mbar, the inert gas flow was between 0.92 and 0.65 L h^‒1^, or related to the membrane area it was $F_{\mathrm{inert},M}^{0}/A_{M}$ = 53 to 38 L m^‒2^ h^‒1^ or $J_{\mathrm{inert}}$ = 2.4 to 1.7 mol m^‒2^ h^‒1^. Yang et al. [19] determined the inert gas flow for a PDMS/ceramic composite membrane at $p_{\mathrm{perm}}$ = 10 mbar and obtained a distinctly higher flux of 7.9 mol m^‒2^ h^‒1^, possibly due to a smaller membrane thickness of only 4 µm. Liang et al. [38] found an even higher inert gas flux of 12 mol m^‒2^ h^‒1^ at $p_{\mathrm{perm}}$ = 3 mbar for a PDMS/polyamide composite membrane.

The here observed area‐specific inert gas flow is quite low but high enough to significantly influence the separation process; inert gas passing through the membrane dilutes the ethyl acetate at the permeate side and thus reduces $x_{\mathrm{EA},\mathrm{down}}\left(r\right)$ (see Equation 13) which in turn increases the flux of ethyl acetate (cf. Equation 10). At $p_{\mathrm{perm}}$ = 10 mbar and feed gas flows of $F_{\mathrm{feed}}^{0}$ = 13.5 to 232.2 L h^‒1^, the inert gas flow amounted to 6.8% to 0.4% of the feed gas.

The literature reports on swelling of the polymeric matrix of MMM due to the absorption of the transported compound, causing the microscopic defects in the membrane to narrow and the inert gas flow to become smaller [39, 40]. To check whether this effect also occurs with the membrane used here, a gas flow with varying amounts of ethyl acetate was supplied to the module and the permeate flow was measured with the constant volume/variable pressure method. The permeate gas consisted of a mixture of inert gas and ethyl acetate, that is, $F_{\mathrm{perm}}^{0}=F_{\mathrm{inert},M}^{0}+F_{\mathrm{EA},M}^{0}$ with $F_{\mathrm{EA},M}^{0}=J_{\mathrm{EA}}\cdot A_{M}\cdot V_{m}^{0}$. The flow of ethyl acetate became gradually smaller at a decreasing permeate pressure, and the permeate gas flow more and more approached the value of $F_{\mathrm{inert},M}^{0}$ which means that $F_{\mathrm{inert},M}^{0}$ was not influenced by the presence of ethyl acetate in the feed gas (Supporting Information 3).

#### 3.3.2 Separation of Ethyl Acetate by a Single Membrane Module

The separation experiments were performed under conditions as given in Figure 2. Then, the evaluation parameters defined in Equations (1) to (4) were determined as described in Section 3.3.1 based on $x_{\mathrm{EA},\mathrm{feed}}$, $x_{H_{2}O,\mathrm{feed}}$, $x_{\mathrm{EA},\mathrm{ret}}$ and $x_{H_{2}O,\mathrm{ret}}$ which were quasi‐continuously measured by MS.

The evaluation parameters were significantly influenced by the permeate pressure $p_{\mathrm{perm}}$ (Figure 2); all parameters were maximal at the lowest pressure, but steeply decreased with the increase of $p_{\mathrm{perm}}$ and asymptotically approached zero. Similar results were obtained at VOC separation from nitrogen by a PDMS composite membrane [26]. A high pressure gradient is essential for a successful separation of ethyl acetate but, on the other hand, a low $p_{\mathrm{perm}}$ means a high energy demand for generating the vacuum. Therefore, a compromise must be found between a high separation efficiency and an acceptable energy consumption; such a compromise can only be determined by economic analyses of the separation process, which was not in the scope of this work.

The ester flux $J_{\mathrm{EA}}$ increased with both an increasing $x_{\mathrm{EA},\mathrm{feed}}$ and an increasing $F_{\mathrm{feed}}^{0}$ (Figure 2G–I). This observation can be explained by the fact that a rise in $x_{\mathrm{EA},\mathrm{feed}}$ and/or $F_{\mathrm{feed}}^{0}$ increases the driving force for ester transport through the membrane. The maximum ester flux was found to be $J_{\mathrm{EA}}$ = 4.2 mol m^‒2^ h^‒1^ at $p_{\mathrm{perm}}$ = 10 mbar, $x_{\mathrm{EA},\mathrm{feed}}$ = 0.045 L L^‒1^ and $F_{\mathrm{feed}}^{0}$ = 60.5 L h^‒1^ (Figure 2I). Yang et al. [19] determined the flux of ethyl acetate through a PDMS/ceramic composite membrane at $p_{\mathrm{perm}}$ = 10 mbar and a varied ester content in the feed gas and obtained $J_{\mathrm{EA}}$ = 15.6 mol m^‒2^ h^‒1^ at $x_{\mathrm{EA},\mathrm{feed}}$ = 0.045 L L^‒1^; the higher flux results from a low thickness of the active separation layer of only 4 µm.

The separation yield $Y_{\mathrm{EA}}$ increased with a decreasing feed gas flow (Figure 2A–C). The smaller $F_{\mathrm{feed}}^{0}$, the longer the residence time of the feed gas in the module, which intensified the separation. The ester content in the feed $x_{\mathrm{EA},\mathrm{feed}}$, on the other hand, had only a minor influence on $Y_{\mathrm{EA}}$. The maximum separation yield of $Y_{\mathrm{EA}}$ = 0.90 L L^‒1^ was observed at $p_{\mathrm{perm}}$ = 10 mbar and $F_{\mathrm{feed}}^{0}$ = 13.5 L h^‒1^ which means that 90% of the supplied ethyl acetate was separated by a single module. A small feed gas flow in relation to the membrane area ($F_{\mathrm{feed}}^{0}$/$A_{M}$) is, however, economically inefficient.

The separation factor $\alpha_{\mathrm{EA}}^{*}$ is often used for the evaluation of separation processes [19, 24, 25]. The separation factor increased when $F_{\mathrm{feed}}^{0}$ was increased, but the value of $x_{\mathrm{EA},\mathrm{feed}}$ had virtually no effect on $\alpha_{\mathrm{EA}}^{*}$. A maximum value of $\alpha_{\mathrm{EA}}^{*}$ = 60 was obtained for the highest feed gas flow applied. Yang et al. [19] separated ethyl acetate from nitrogen, containing various amounts of the ester by a PDMS/ceramic composite membrane at $p_{\mathrm{perm}}$ = 10 mbar and observed a dependence of the separation factor on $x_{\mathrm{EA},\mathrm{feed}}$, but only at low $x_{\mathrm{EA},\mathrm{feed}}$ values, while at higher $x_{\mathrm{EA},\mathrm{feed}}$ values the separation factor was uniformly $\alpha_{\mathrm{EA}}^{*}$ = 42.

The enrichment factor $E_{\mathrm{EA}}$ quantifies the enrichment of ethyl acetate in the permeate and delivers information on how the ester is concentrated in the permeate. The increase in $F_{\mathrm{feed}}^{0}$ raised the flux of ethyl acetate through the membrane and thus increased $x_{\mathrm{EA},\mathrm{perm}}$ and $E_{\mathrm{EA}}$. The increase in $x_{\mathrm{EA},\mathrm{feed}}$ heightens $J_{\mathrm{EA}}$ and thus should increase $E_{\mathrm{EA}}$, but on the other hand, $E_{\mathrm{EA}}$ is defined as the $x_{\mathrm{EA},\mathrm{perm}}/x_{\mathrm{EA},\mathrm{feed}}$ quotient so that increasing $x_{\mathrm{EA},\mathrm{feed}}$ values finally diminished $E_{\mathrm{EA}}$. A maximum value of $E_{\mathrm{EA}}$ = 44 L L^‒1^ was received for the highest feed gas flow in combination with the smallest $x_{\mathrm{EA},\mathrm{feed}}$ applied.

A mathematical model was derived in Section 3.2 to describe the separation of ethyl acetate by a circular membrane module. All parameters in this model were determined independently with the exception of the membrane permeability $P_{\mathrm{EA},M}$. This parameter was the only one which was identified by adaptation of the simulated to the measured data, yielding $P_{\mathrm{EA},M}$ = 4.7 × 10^‒7^ mol s^‒1^ m^‒1^ bar^‒1^. Model simulations are depicted in Figure 2 as solid lines together with the measured data. The model describes the separation process quite well. Larger deviations were only observed in separation processes with the lowest $x_{\mathrm{EA},\mathrm{feed}}$ value tested (Figure 2A,D,G,J) which is explainable by higher measuring errors at low ester concentrations in the gas flows. Deviations became also visible at permeate pressures of 200 and 500 mbar due to higher measuring errors at low ester fluxes, but these deviations are without practical significance because such permeate pressures lead to poor separation anyway.

#### 3.3.3 Separation of Ethyl Acetate by Two Serial Membrane Modules

**FIGURE 3:**
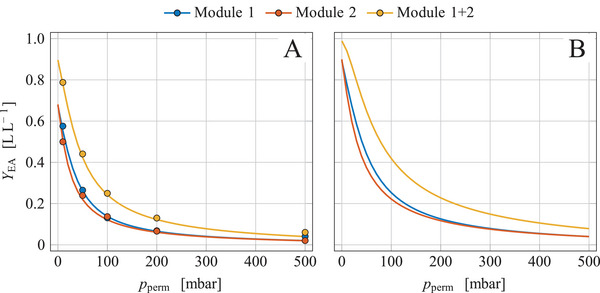
Separation yield of two identical membrane modules connected to each other in series at various permeate pressures using artificial exhaust gas; the artificial feed gas based on air contained a defined amount of ethyl acetate of $x_{\mathrm{EA},\mathrm{feed}}$ = 0.025 L L^‒1^; the feed‐gas flow was set to (A) $F_{\mathrm{feed}}^{0}$ = 60.5 L h^‒1^ or (B) $F_{\mathrm{feed}}^{0}$ = 30.7 L h^‒1^; $Y_{\mathrm{EA}}$ was determined for the single modules and for their combination as indicated in the legend; symbols mark data based on measurements while solid lines represent model simulations; conditions and parameters: $x_{H_{2}O,\mathrm{feed}}$ = 0.013 L L^−1^, $T_{M}$ = 40°C, $p_{\mathrm{feed}}$ = 998.5 mbar, $P_{\mathrm{EA},M}$ = 4.7·10^‒7^ mol s^‒1^ m^‒1^ bar^‒1^, $\zeta_{M,1}^{*}$ = 6.0·10^14^, $\zeta_{M,2}^{*}$ = 6.8·10^14^.

In biotechnological processes, the gas flow is not a freely adjustable parameter but is determined by the aeration of the actual cultivation process. At cultivation of *K. marxianus* DSM 5422 in 1 L DWP‐based medium, the stirred reactor has been aerated with 1 vvm or 60 L h^‒1^ air under standard conditions [8]. If ethyl acetate were separated from this flow with a single membrane module at $p_{\mathrm{perm}}$ = 10 mbar, a separation yield of only 0.53 to 0.60 L L^‒1^ would be achieved, depending on $x_{\mathrm{EA}}$ in the exhaust gas (Figure 2A–C). It was intended to increase the separation yield by using two membrane modules connected in series as shown in Figure 1A. Separation of ethyl acetate from artificial exhaust gas with two serial modules was exemplarily tested under conditions as given in Figure 3. The measurements and calculations were performed as explained in Section 3.3.1.

The separation yields of the individual modules $Y_{\mathrm{EA},1}$($p_{\mathrm{perm}}$) and $Y_{\mathrm{EA},2}$($p_{\mathrm{perm}}$) were quite similar, but the separation yield of the combined membrane unit was distinctly larger (Figure 3A); for example, at $p_{\mathrm{perm}}$ = 10 mbar, $Y_{\mathrm{EA},1}$ = 0.582 L L^‒1^, $Y_{\mathrm{EA},2}$ = 0.502 L L^‒1^, and $Y_{\mathrm{EA},1+2}$ = 0.793 L L^‒1^. The model calculations (solid lines in Figure 3A) correspond well with the measured data.

The separation yield of a membrane unit consisting of two or more modules arranged in series is estimated with the following equation (Supporting Information 4):

(19) $$Y_{\mathrm{EA},1\text{…}n}=1-\prod_{i=1}^{n}\left(1-Y_{\mathrm{EA},i}\right)$$

For two modules, one obtains $Y_{\mathrm{EA},1+2}=1-\left(1-Y_{\mathrm{EA},1}\right)\cdot \left(1-Y_{\mathrm{EA},2}\right)$. The separation yield of the membrane unit could be further increased by adding more modules, but the inert gas flow would also increase, which dilutes the permeate and makes ethyl acetate condensation more difficult.

Although the separation yield significantly increased with two modules, the value of $Y_{\mathrm{EA},1+2}$ = 0.793 L L^‒1^ was unsatisfactory for practical applications. A reduction in the aeration of the bioreactor from 60 to just 30 L h^‒1^ was therefore intended. Model simulations with $F_{\mathrm{feed},1}^{0}$ = 30.7 L h^‒1^ and $x_{\mathrm{EA},\mathrm{feed},1}$ = 0.025 L L^‒1^ clearly showed that halving the gassing rate of the bioreactor will increase the separation yield to $Y_{\mathrm{EA},1+2}$ = 0.942 L L^‒1^ (Figure 3B).

### 3.4 Separation of Ethyl Acetate from Real Exhaust Gas of a Bioreactor

The separation of microbially synthesized ethyl acetate from the exhaust gas of a bioreactor was studied using the setup shown in Figure 1B. Measurements and model simulations in Section 3.3 demonstrated that the separation of ethyl acetate from the exhaust gas of a bioreactor aerated with 60 L h^‒1^ as usual [8] is ineffective when only using a single membrane module (estimated separation yield below 0.6 L L^−1^). Simulations demonstrated that the combination of two identical modules in series and the reduction of the gas flow to 30 L h^−1^ let expect a separation yield of $Y_{\mathrm{EA}}$ = 0.942 L L^−1^ (Figure 3B). The experiment was therefore performed under these conditions.

#### 3.4.1 Microbial Synthesis of Ethyl Acetate

**FIGURE 4:**
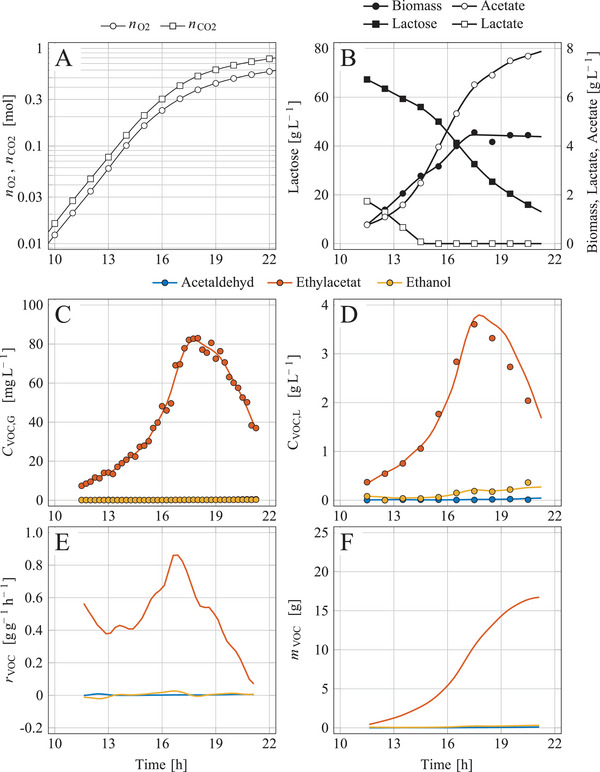
Integrated process of microbial synthesis of ethyl acetate as a combination of aerobic batch cultivation and ISPR using membrane separation and condensation; (A) cumulatively consumed oxygen and formed CO_2_, (B) lactose, biomass, acetate and lactate concentration, (C) VOC concentrations in the exhaust gas measured by GC and MS, (D) VOC concentrations in the liquid phase, (E) biomass‐specific reaction rates of VOC synthesis, (F) masses of synthesized VOCs during the aerobic batch cultivation of *K. marxianus* DSM 5422 in a stirred bioreactor using 1 L DWP^–Fe^ medium; cultivation at 1200 rpm, 40°C, pH 5.1, aeration with 30 L h^−1^ given for standard conditions.

The microbial production of ethyl acetate with *K. marxianus* DSM 5422 was performed under conditions given in the caption of Figure 4. Ester synthesis has been induced by limiting yeast growth due to a lack of iron [5, 6, 7, 10, 29, 41, 42, 43, 44, 45, 46, 47, 48, 49], copper [47], or oxygen [29, 44, 50, 51, 52, 53]. Regarding the mechanism of the ester synthesis, it is referred to [41, 46, 48, 54, 55]. Here, the iron limitation was the inducer of ester formation.

Next, the general course of aerobic batch cultivation is discussed (Figure 4). The oxygen cumulatively consumed and the CO_2_ cumulatively produced (Figure 4A) provide information on the growth mode of the cultivated yeasts. Initially, the yeasts grew exponentially ($d\ln(n_{O_{2}})/dt$ and $d\ln(n_{{\mathrm{CO}}_{2}})/dt$ are constant over time), but at times >13 h, the growth rate decreased due to iron limitation. Iron limitation occurred early and only a small amount of biomass was formed (maximum $C_{X}$ = 4.5 g L^‒1^). The early and severe iron limitation seemed surprising since DWP^‒Fe^ medium contains 200 µg L^−1^ iron; however, the iron‐chelating effect of 9.5 g L^−1^ citrate in the medium reduced the bioavailability of iron (for details see [8, 56]).

The sugar was mainly converted into metabolites such as ethyl acetate, acetate, little ethanol, and traces of acetaldehyde (Figure 4B,E,F). Volatile ethyl acetate was quickly stripped from the bioreactor resulting in high ester concentrations in the exhaust gas (Figure 4C). Stripping restricted accumulation of the ester in the culture medium (Figure 4D). However, reducing the aeration rate to 30 L h^‒1^ resulted in slightly higher dissolved‐ester concentrations as usual [8] without reaching critical values [10]. Ethanol stripping was without importance due to its much lower volatility [9, 57] compared to ethyl acetate. Ethanol becomes more volatile and is recoverable by stripping at higher temperatures as it is the case at cultivation of thermophilic bacteria [58].

#### 3.4.2 Separation of Microbially Synthesized Ethyl Acetate

**FIGURE 5:**
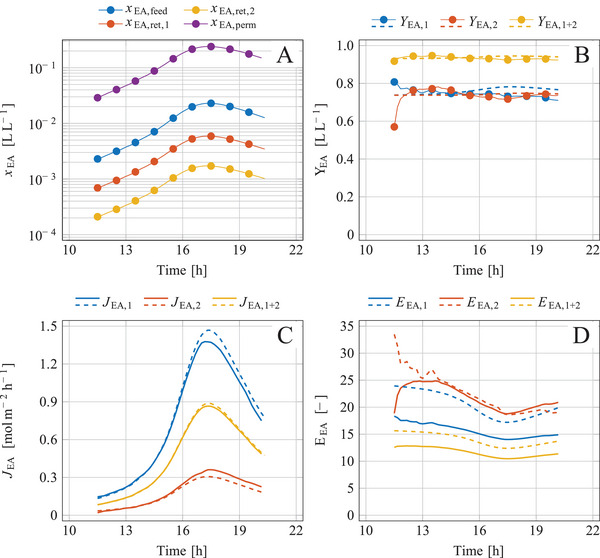
Integrated process of microbial synthesis of ethyl acetate as a combination of aerobic batch cultivation and ISPR using membrane separation and condensation; membrane separation performance during the aerobic batch cultivation shown in Figure 4; the membrane unit consisted of two identical membrane modules connected in series; (A) fraction of ethyl acetate in gas flows, (B) separation yield, (C) area‐specific flux of ethyl acetate through the membrane, and (D) enrichment factor of ethyl acetate, each determined for the two single modules and for the membrane unit; measured data (solid lines) and model simulations (dashed lines); conditions and parameters: $x_{H_{2}O,\mathrm{feed}}$ = 0.013 L L^−1^, $T_{M}$ = 40°C, $p_{\mathrm{feed}}$ = 996.2 mbar, $p_{\mathrm{perm}}$ = 10 mbar, $P_{\mathrm{EA},M}$ = 4.7·10^‒7^ mol s^‒1^ m^‒1^ bar^‒1^, $\zeta_{M,1}^{*}$ = 6.0·10^14^, $\zeta_{M,2}^{*}$ = 6.8·10^14^.

ISPR of the ester was performed by connecting the bioreactor with the membrane unit at a process period from 11.5 to 20.5 h. The membrane unit consisted of two identical modules connected in series (Figure 1B), equipped with MMM having a separation area of each 0.0172 m^2^. The exhaust‐gas flow (=$F_{\mathrm{feed},1}^{0}$) determined as described in [9] slightly varied from 30.16 to 31.04 L h^‒1^. The quasi‐continuously measured fractions of ethyl acetate in the feed and the retentate of both modules (Figure 5A) were used to balance the ester for the single modules and for the membrane unit as a whole (Section 3.1). The high temporal resolution of the measurements enabled precise determination of $F_{\mathrm{perm}}^{0}$ and $x_{\mathrm{EA},\mathrm{perm}}$ despite the dynamic variation of the ester content in the exhaust gas (Figure 5A).

During the separation period, 16.28 g ethyl acetate was microbially formed (Figure 4F), and 14.60 g ester was stripped and fed to the membrane unit; the difference of 1.68 g was ester already synthesized but not yet stripped (Figure 4D). The content of ethyl acetate in the gas was distinctly reduced after passing the two modules (Figure 5A). The degree of separation was similar for both modules (Figure 5B) as already observed with artificial exhaust gas (Figure 3). The separation yields were not noticeably influenced by the ester content in the feed gas (cf. Figure 5A with Figure 5B). The separation yields averaged over time were $Y_{\mathrm{EA},1}$ = 0.749 L L^‒1^, $Y_{\mathrm{EA},2}$ = 0.745 L L^‒1^, and $Y_{\mathrm{EA},1+2}$ = 0.936 L L^‒1^. The concept of increase $Y_{\mathrm{EA}}$ by using two modules in series and halving the gas flow was very successful. The measured separation yields nearly matched the model‐based prediction of $Y_{\mathrm{EA},1+2}$ = 0.942 L L^‒1^ (Figure 3B). An absolute amount of 13.67 g ester was separated by the membrane unit. The separation yield could have been further increased to 0.984 L L^‒1^ by using a third membrane module; however, an additional module would increase the inert gas flow and thus more dilute the ethyl acetate in the permeate and complicate its condensation.

The fluxes of ethyl acetate $J_{\mathrm{EA},1}$, $J_{\mathrm{EA},2}$ and $J_{\mathrm{EA},1+2}$ followed the kinetics of the ester in the exhaust gas (cf. Figure 5A,C). The higher the ester content was, the larger the fluxes became. The flux $J_{\mathrm{EA},2}$ was distinctly smaller than flux $J_{\mathrm{EA},1}$ (Figure 5C) which is explained by the lower ester content in the feed of the second module with the consequence of a lower driving force for the ester transport. The measured data and the model calculations agree well.

The enrichment factor $E_{\mathrm{EA}}$ provides information about the concentration of ethyl acetate in the permeate compared to the feed. For engineering purposes, $E_{\mathrm{EA}}$ is easier to interpret than parameter $\alpha_{\mathrm{EA}}^{*}$. The enrichment was calculated for both modules separately and for the membrane unit as a whole (Figure 5D). $E_{\mathrm{EA},1+2}$ was lower than the enrichment of the individual modules $E_{\mathrm{EA},1}$ and $E_{\mathrm{EA},2}$. This observation is explained by the inert gas flow, which dilutes the ethyl acetate in the permeate; the higher the number of modules, the more inert gas dilutes the permeate and the smaller the enrichment factor becomes. Model calculations of the enrichment factor distinctly differed from the measured data (Figure 5D) which is reasoned by water passing through the membrane that was taken into account at balancing (solid lines) but ignored at simulation (dashed lines). Water permeating through the membrane dilutes the ester in the permeate in addition to the inert gas and thus reduces $E_{\mathrm{EA}}$ based on measured data. This effect was more pronounced in the first module, as the water content in the feed of the second module was already greatly reduced (confirmed by measurements as shown in Supporting Information 5).

The content of ethyl acetate in the permeate gas is important for the condensation of the ester. The lower $x_{\mathrm{EA},\mathrm{perm}}$, the lower the temperature at which the ester starts to condense. A high ester content in the permeate gas is therefore desirable.

The combined permeate gas flow of both modules passed through two cold traps connected in series to condense the volatiles. Liquid nitrogen was not suitable as a coolant (water vapor formed ice at the inlet of the first trap and blocked the gas flow after a short time), but a mixture of acetone and solid CO_2_ having a temperature of ‒78°C worked well.

Condensate was only formed in the first cold trap which consisted of two phases, an upper organic and a lower aqueous phase. Analysis of the condensate revealed the following masses: 11.10 g ethyl acetate, 2.80 g water, and 0.028 g ethanol; hence, 99.75% of the organic fraction was ethyl acetate. Separation of condensed ethyl acetate, ethanol, and water is possible by distillation processes [59, 60, 61, 62].

Comparison of the permeated and condensed ethyl acetate (13.66 vs. 11.10 g) yielded a difference of 2.56 g, which represents ethyl acetate that was separated but not condensed in the cold traps. The relatively high ester losses are explained by the low pressure of 10 mbar during condensation. Condensation on the pressure side instead of the suction side would have led to much better condensation, but the vacuum pump used did not allow this setup.

## 4 Concluding Remarks

The presented study demonstrates the ISPR of microbially produced ethyl acetate from the exhaust gas of a bioreactor by membrane technology for the first time. The yeast *K. marxianus* DSM 5422 effectively converts sugar‐rich waste of the food industry to this ester in an aerobic process, whereby the synthesized ester is quickly stripped with the exhaust gas. The stripped ethyl acetate was effectively separated from the exhaust gas by a MMM made of Silicalite‐1 and polydimethylsiloxane. The separation was highly influenced by the flow and ester content of the exhaust gas as well as by the permeate pressure. Under optimized conditions, 93.6% of the stripped ester was separated from the exhaust gas, and 99.75% of the condensed organic compounds consisted of ethyl acetate. A mathematical model described the separation process quite well and supported the design of the main experiment.

Future improvements are to be achieved by optimizing the membrane to reduce the inert gas flux, increase $x_{\mathrm{EA},\mathrm{perm}}$ and thus facilitate ester condensation. Both the fraction of the condensed ester and the condensation temperature will be increased through condensation at the pressure side of the vacuum pump. Moreover, the energy consumption for membrane separation and condensation will be determined and then compared with the energy requirement for direct condensation of the ester from the exhaust gas.

This study shows that ethyl acetate microbially produced in an aerobic bioprocess and stripped with the exhaust gas can be effectively separated by membrane technology and condensed to obtain ethyl acetate in high purity.

## Nomenclature

 

$A_{M}$ (m^2^): surface area of the membrane
$C_{\mathrm{EA},M,j}$ (kg m^−3^): concentration of ethyl acetate in the membrane material at the membrane surface, i.e., at the membrane‐gas interface, at position j
$C_{\mathrm{VOC},j}$ (kg m^−3^): concentration of a volatile organic compound in phase j
$D_{\mathrm{EA},M}$ (m^2^ s^−1^): diffusion coefficient of ethyl acetate in the membrane material
$E_{\mathrm{EA}}$ [‒]: enrichment factor of ethyl acetate at membrane separation
$F_{j}^{0}$ (m^3^ s^−1^): gas flow at position j at standard conditions
$F_{i,j}^{0}$ (m^3^ s^−1^): gas flow of compound i at position j at standard conditions
$H_{\mathrm{EA},M/G}$ (mol m^−3^ bar^−1^): Henry coefficient of ethyl acetate for the membrane/gas system
$J_{i}$ (mol m^−2^ s^−1^): area‐specific flux of component i through the membrane
$n_{i}$ (mol): cumulatively consumed oxygen or formed CO_2_
$p^{0}$ (bar): pressure at standard conditions (1013.25 mbar)
$P_{\mathrm{EA},M}$ (mol s^‒1^ m^‒1^ bar^‒1^): permeability coefficient of the membrane for ethyl acetate
$p_{i,j}$ (bar): partial pressure of compound i in the gas at position j
$p_{j}$ (bar): absolute pressure at position j
r (m): radial position on the circular membrane
$r_{\max}$ (m): radius of the circular membrane
$R_{\mathrm{VOC}}$ (g L^‒1^ h^‒1^): volume‐specific formation rate of a volatile organic compound
$r_{\mathrm{VOC}}$ (g g^‒1^ h^‒1^): biomass‐specific formation rate of a volatile organic compound
$T^{0}$ (K): temperature at standard conditions (273.15 K)
$T_{M}$ (°C): temperature of the membrane
$V_{m}^{0}$ (L mol^−1^): molar gas volume at standard conditions
$x_{i,j}$ (L L^−1^): volume fraction of compound i in the gas flow j
$Y_{\mathrm{EA}}$ (−): separation yield of ethyl acetate at membrane separation
$z_{M}$ (m): thickness of the active membrane layer

## Greek symbols

$\alpha_{\mathrm{EA}}^{*}$ (−): separation factor for ethyl acetate relative to all other components
$\zeta_{M}^{*}$ (−): pressure loss coefficient of the membrane

## Indices

down: downstream side of the membrane
EA: dompound ethyl acetate
feed: feed gas of the membrane module
G: gas phase
H_2_O: compound Water
inert: inert gas consisting of N_2_, O_2_ and/or CO_2_
L: liquid phase
M: membrane
perm: permeate gas of the membrane module
ret: retentate gas of the membrane module
up: upstream side of the membrane
VOC: volatile organic compounds

## Conflicts of Interest

The authors declare no conflicts of interest.

## Supporting information



Supplementary information

Supplementary information

Supplementary information

Supplementary information

Supplementary information

## Data Availability

The data that support the findings of this study are available from the corresponding author upon reasonable request.
