# Supplementary material for: In situ Product Recovery of Microbially Synthesized Ethyl Acetate from the Exhaust Gas of a Bioreactor by Membrane Technology
Source: Eng Life Sci. 2024 Sep 30;24(12):e202400041. doi: 10.1002/elsc.202400041 (PMC11620624; doi:10.1002/elsc.202400041)
Supplement: Supplementary file 1 — Supplementary information [file ELSC-24-e202400041-s003.pdf]

Supporting Information 1:

Membrane preparation and membrane module construction

In situ product recovery of microbially synthesized ethyl acetate from the exhaust gas of a bioreactor by membrane technology

Andreas Hoffmann, Alexander Franz, Christian Löser, Thomas Hoyer, Marcus Weyd, Thomas Walther

Used symbols are listed in the main part of the work or are explained here in the text.

Membrane preparation

Mixed matrix membranes consist of separation-active particles which are embedded in a polymeric matrix. Here, Silicalite-1 particles were combined with polydimethylsiloxane (PDMS). The active separation layer was connected with the support fleece by a porous intermediate layer with a thickness of about 80 µm. For the preparation of the active separation layer, aluminum-free Silicalite-1 powder was fractionated by sieving to a particle size of ≤ 36 µm. This particle fraction was dried at 150 °C for 6 h. 60 g of the dried Silicalite-1 powder were dispersed in 50 g liquid PDMS mixture. In detail, a 0.5/0.5 (m/m) blend of linear PDMS with Si-H or vinyl groups at the ends of their molecules in combination with a platinum catalyst was prepared. An amount of 25 g butan-2-ol was added to the Silicalite-1-PDMS mixture to assist the dispersion process. The prepared mixture was then applied using the doctor blade coating system. The evaporation of the butan-2-ol and the thermal cross-linking was carried out at 150 °C for 10 min. The doctor blade coating system was adjusted in such a manner that the active separation layer had a thickness of 10 µm. The surface of the produced membrane is shown in Figure S1.1.

The membrane produced in this way was visually inspected for possible production defects which were marked in color. Regions without defects were used for preparing circular membrane disks with a diameter of 154 mm. The membrane disk was mounted into a housing shown below and formed the so-called membrane module.

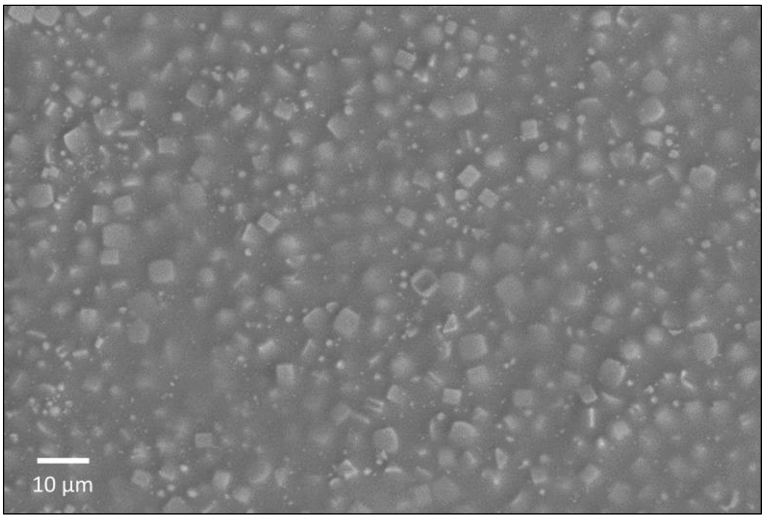

Figure S1.1 Membrane surface consisting of Silicalite-1 embedded in polydimethylsiloxane (picture prepared by scanning electron microscopy).

Membrane module construction

The membrane module consists of the membrane, a sintered plate supporting the membrane, an EPDM rubber seal and the housing (Figure S1.2).

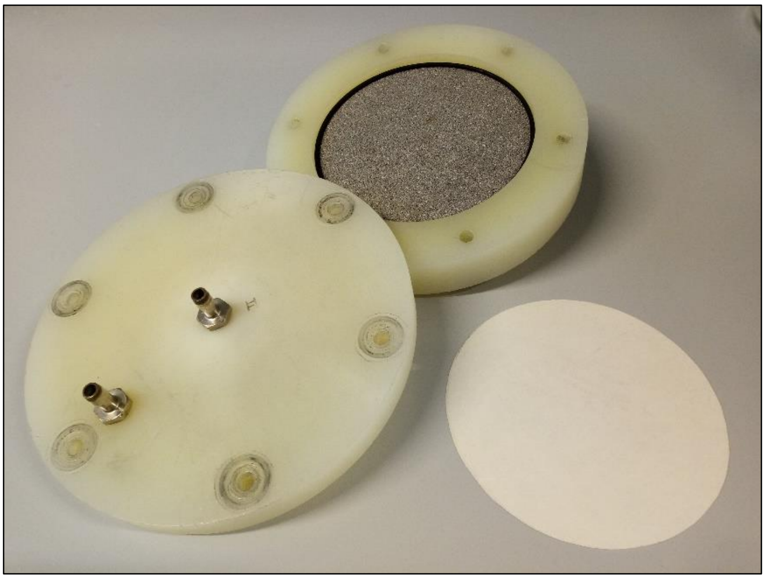

Figure S1.2 Membrane module consisting of the membrane, the upper and lower part of the housing, and a sintered plate including the EPDM rubber seal both located in the lower part of the housing.

The membrane with a diameter of 154 mm is gas-tight against the upper part of the housing by the EPDM rubber seal. The rubber seal covers the outermost region of the membrane so that the effective diameter is reduced to 148 mm, resulting in a separation area of A_M = 0.01720 m^2. The membrane lies on a porous plate, with its fleece side oriented to the plate and its active separation layer oriented to the feed gas. The porous plate is made of sintered V4A steel with a diameter of 153 mm, a thickness of 8 mm, a porosity of 0.495 L L^{-1} and an inner gas volume of 72.8 mL.

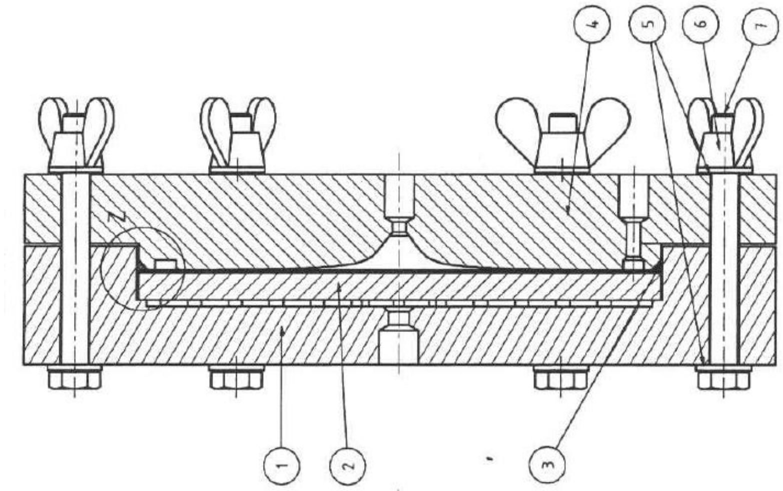

Figure S1.3 Membrane module used for separation of ethyl acetate from a feed gas flow which is divided into the permeate gas flow and the retentate gas flow; ① = lower part of housing, ② = porous plate, ③ = EPDM rubber seal, ④ = upper part of housing, ⑤ = washers, ⑥ = wing nuts, ⑦ = M8 screws; (Scale = 1:2).

The module housing with an outer diameter of 220 mm consists of an upper part, a lower part and six M8 screws. The upper and lower parts are made of polypropylene and were manufactured by turning and milling. The upper part has a central opening for supplying the feed gas and a ring-shaped collecting channel in the outer region of the housing, which is connected to an opening for discharging

the retentate gas. The lower part of the housing accommodates the sintered plate, which rests on a channel system (consisting of intersecting radial and concentric channels) that guides the permeate gas to a central opening. This channel system contains a gas volume of 15.2 mL, so that the total gas volume at the permeate side in the sintered plate and channel system is 88.0 mL.

As the separation process is influenced by the temperature, the module was housed in a polystyrene foam box in which the temperature was controlled to 40 °C.

The feed gas (with the flow F_{feed}) supplied to the membrane module flows through a ring gap between the upper part of the housing and the membrane and leaves the module via the retentate port. The height of the ring gap through which the gas flows is determined by the rotation-symmetrical profile on the inside of the upper part of the housing (Figure S1.3). The height of the flow channel decreases as the radius increases according to the following relationship:

$$h_C = \frac{k}{r} \qquad r_{min} \leq r \leq r_{max} \tag{S1.1}$$

The design parameter k is determined by the profile in the upper part of the housing and is k = 32.25 mm^2. The minimum and maximum radii are r_{min} = 3 mm and r_{max} = (148 mm)/2 = 74 mm. The cross-section of the ring gap through which the gas flows is determined by the height and width of the gap, whereby the width corresponds to the circumference at position r (b_C = $2\pi \cdot r$). The following therefore applies:

$$A_C = h_C \cdot b_C = 2\pi \cdot k \tag{S1.2}$$

According to equation (S1.2), the cross-sectional area through which the gas flows is independent of the radius r and is uniformly A_C = 202.6 mm^2 for all radii. The averaged flow velocity of the gas in the ring gap results from the continuity equation (neglecting the reduction of the gas flow due to the permeation of part of the gas through the membrane):

$$\overline{\omega} = \frac{F_{feed}}{A_C} = \frac{F_{feed}}{2\pi \cdot k} \tag{S1.3}$$

According to equation (S1.3), the mean flow velocity of the gas in the ring gap is also independent of the radius. The average contact time of the flowing feed gas with the membrane is calculated from the mean flow velocity and the distance moved. To calculate the total residence time, the total distance covered by the feed gas in the ring gap is used:

$$\tau = \frac{r_{max} - r_{min}}{\overline{\omega}} = 2\pi \cdot k \cdot \frac{r_{max} - r_{min}}{F_{feed}} \tag{S1.4}$$

In the experimental investigations, the feed gas flow ranged from F_{feed} = 14.1 to 282 L h^{-1}, resulting in average residence times of 3.67 s for the smallest gas flow and 0.184 s for the largest gas flow.

Finally, the pressure loss in the feed gas flow was measured as it passed through the ring gap. As expected, the pressure loss increased with the volume flow, but even with the largest gas flow of F_{feed} = 282 L h^{-1}, the pressure loss was only 5 mbar and thus insignificant.

The expected permeate gas flows are so low that it did not appear necessary to analyze the flow regime on the permeate side of the membrane.
