# Supplementary material for: In situ Product Recovery of Microbially Synthesized Ethyl Acetate from the Exhaust Gas of a Bioreactor by Membrane Technology
Source: Eng Life Sci. 2024 Sep 30;24(12):e202400041. doi: 10.1002/elsc.202400041 (PMC11620624; doi:10.1002/elsc.202400041)
Supplement: Supplementary file 2 — Supplementary information [file ELSC-24-e202400041-s001.pdf]

Supporting Information 2:

Mass spectrometry

In situ product recovery of microbially synthesized ethyl acetate from the exhaust gas of a bioreactor by membrane technology

Andreas Hoffmann, Alexander Franz, Christian Löser, Thomas Hoyer, Marcus Weyd, Thomas Walther

Used symbols are listed in the main part of the work or are explained here in the text.

The composition of the exhaust gas of the bioreactor was analyzed by mass spectrometry using a GAM 2000 mass spectrometer (InProcess Instruments, Bremen, Germany) which was combined with a specific sampling station. The analysis concentrated on determination of the volume fractions of oxygen, carbon dioxide, water and the VOCs ethyl acetate and ethanol.

Specific construction

The GAM 2000 mass spectrometer and the construction of the user-specific sampling station is shown in Figure S2.1. The individual parts of the system are named in the caption of Figure S2.1, and their individual functions are explained in connection with the operation of the mass spectrometer.

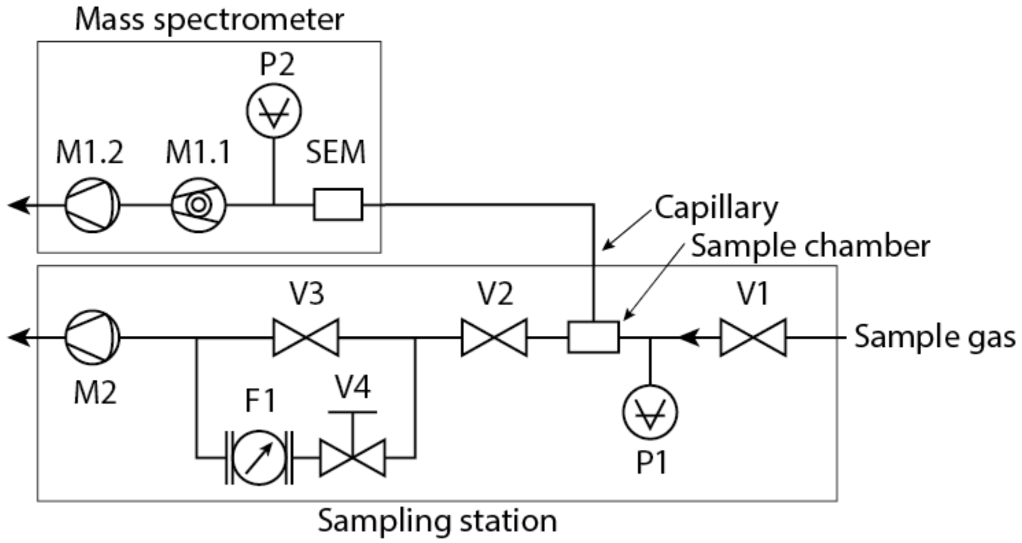

Figure S2.1 Simplified scheme of the GAM 2000 mass spectrometer. The gaseous sample is transported to the sample chamber using the gas sampling station and transferred via a capillary to the mass spectrometer. V1, V2, V3 are magnetic valves and V4 is a manual valve. P1 and P2 are pressure gauges. M1.1 and M1.2 are the turbo pump and the fore diaphragm pump of the mass spectrometer, and M2 is the diaphragm pump of the sampling station. F1 is a flow meter. The sample chamber holds a gas volume of approx. 10 mL.

Operation

The exhaust gas of the bioreactor was partially dehumidified by cooling down to 11 °C to avoid condensation of water within the analysis equipment. The dehumidified exhaust gas was then fed to the sampling station. A T-connector was used at the inlet of the sampling station for discharging

excess exhaust gas (not shown in Figure S2.1). To minimize gas contamination with the previously analyzed sample, the following procedure was applied:

- Purge sample chamber with sample gas: Valve V4 is permanently closed, valve V1 is closed, valves V2 and V3 are opened, and M2 is turned on for 5 s, resulting in a total pressure of less than 10 mbar within the sample chamber. Valve V2 is then closed and the gas inlet valve V1 is opened to fill the sample chamber with sample gas. The gas inlet valve V1 is then closed. This flushing process is carried out twice.
- Sample transfer to the mass spectrometer: Gas from the sample chamber is constantly transferred to the mass spectrometer by a pressure gradient over the heated capillary. As the capillary is quite long (1.8 m), the gas transport from the sample chamber to the mass spectrometer takes around 2 min.
- Sample analysis: The gas sample is analyzed by a secondary electron multiplier (SEM) detector using the following parameters for each mass-to-charge ratio (m/z) to be analyzed: a pre-settling time of 120 ms, a settling time of 50 ms, and an integration time of 70 ms.

This procedure was repeated for each sample point. The described procedure makes it possible to quantify the composition of gases from different sampling points without cross-contamination.

Calibration

The mass spectrometer was calibrated for several exhaust gas compounds, including argon (Ar), nitrogen (N_2), oxygen (O_2), carbon dioxide (CO_2), water, ethyl acetate, and ethanol. Ar was used as an internal standard. A mixture containing 0.3001 L L^{-1} N_2, 0.3001 L L^{-1} O_2 and argon (relative uncertainty: ±0.1 %, Linde, Germany) was used for calibration of N_2 and O_2. CO_2 calibration was performed using a mixture of 0.3000 L L^{-1} CO_2 and argon (relative uncertainty: ±0.1 %, Linde, Germany).

For the calibration of the mass spectrometer for water, ethyl acetate and ethanol, a strategy was used in which argon 5.0 (Nippon Gases, Germany) was passed through a bubble column containing the compound in question at a defined temperature. The composition of the leaving gas flow was calculated from the saturation vapor pressure depending on temperature using a modified Antoine equation: $p_i = A_i^* \cdot 10^{(B_i^*/(C_i^*+T))}$. The following parameters were received when transforming the data from https://www.ddbst.com/: $A_{H_2O}^*$ = 1.571·10^{10} Pa, A_{EA}^* = 1.685·10^9 Pa, A_{EtOH}^* = 2.133·10^{10} Pa, $B_{H_2O}^*$ = –1730.63 K, B_{EA}^* = –1244.95 K, B_{EtOH}^* = –1642.89 K, $C_{H_2O}^*$ = –39.7 K, C_{EA}^* = –55.2 K, and C_{EtOH}^* = –42.85 K.

The calibration gases were passed continuously through the sample chamber and analyzed with the GAM 2000. This was achieved by opening V1, V2 and V4 and closing V3. The gas flow rate was set to a value of 40 L h^{-1} via flow meter F1 (Figure S2.1).
