# Supplementary material for: In situ Product Recovery of Microbially Synthesized Ethyl Acetate from the Exhaust Gas of a Bioreactor by Membrane Technology
Source: Eng Life Sci. 2024 Sep 30;24(12):e202400041. doi: 10.1002/elsc.202400041 (PMC11620624; doi:10.1002/elsc.202400041)
Supplement: Supplementary file 3 — Supplementary information [file ELSC-24-e202400041-s005.pdf]

Supporting Information 3:

Inert gas flow through the membrane

In situ product recovery of microbially synthesized ethyl acetate from the exhaust gas of a bioreactor by membrane technology

Andreas Hoffmann, Alexander Franz, Christian Löser, Thomas Hoyer, Marcus Weyd, Thomas Walther

Used symbols are listed in the main part of the work or are explained here in the text.

Calculation of flux J_{inert} and flow $F^0_{inert,M}$

There exists a flux of inert gases (oxygen, nitrogen and carbon dioxide), J_{inert}, which passes through the membrane. This flux influences the separation process since the permeating inert gas dilutes the separated ethyl acetate in the generated permeate gas so that the volume fraction of ethyl acetate in the permeate is reduced ($x_{EA,perm}$ < 1). This $x_{EA,perm}$ reduction also affects the separation process since the flux of ethyl acetate through the membrane, J_{EA}, depends on the gradient of the partial pressure of ethyl acetate across the membrane (for details see main part of this work). The following equation applies to the inert-gas flux through the membrane:

$$J_{inert} = \frac{F^0_{inert,M}}{A_M \cdot V^0_m} \qquad \text{(S3.1)}$$

Herein, $F^0_{inert,M}$ is the absolute volume flow of inert gas through the membrane at standard conditions, A_M is the membrane area, and V^0_m is the molar gas volume at standard conditions. The inert gas flow $F^0_{inert,M}$ depends on the pressure gradient across the membrane. The $F^0_{inert,M}$-Δp dependency was measured for various pressure differences of Δp = $p_{feed} - p_{perm}$ and was described with a model (see below). This model allows the calculation of $F^0_{inert,M}$ and J_{inert} depending on the values of p_{feed} and p_{perm}.

In case of membranes without macroscopic membrane defects, the inert gas only passes through microscopic defects in the polymeric membrane material [Xu et al. 2014]. Then the relationship between $F^0_{inert,M}$ and Δp can be described using Bernoulli's equation for flows in which the flowing medium causes a loss in pressure. Due to the short flow path (the active membrane layer is only 10 µm thick), it is obvious that the pressure loss occurs primarily as an inlet loss:

$$\Delta p = p_{feed} - p_{perm} = \zeta_M \cdot \frac{\rho_{feed}}{2} \cdot \omega_M^2 \qquad \text{(S3.2)}$$

Herein, ζ_M is the pressure loss coefficient, ρ_{feed} is the density of the feed gas under the conditions prevailing at the upper surface of the membrane, and ω_M is the flow velocity of the gas at the inlet of the pores. The conditions at the upper surface of the membrane (temperature T_M and pressure p_{feed}) are valid since the inlet loss develops at this position. There is almost no loss in pressure in the flow from the feed inlet to the retentate outlet, and the retentate outlet is open to the surroundings; therefore, p_{feed} is identical with the ambient pressure p_A.

According to the continuity equation the following is true: $F_{inert,M} = \omega_M \cdot A_M \cdot a_{pores}$. Combination with Eq. (S3.2) after rearrangement gives:

$$F_{inert,M} = A_M \cdot a_{pores} \cdot \left(\frac{2 \cdot (p_{feed} - p_{perm})}{\rho_{feed} \cdot \zeta_M}\right)^{0.5} \tag{S3.3}$$

Herein, a_{pores} describes the area of the pores related to the area of the membrane (given in $m^2\ m^{-2}$). Eq. (S3.3) delivers the absolute inert gas flow at process conditions. Parameter a_{pores} and parameter ζ_M are unknown, which is why they are combined to a new parameter ζ_M^* (with $\zeta_M^* = \zeta_M / a_{pores}^2$). Furthermore, flow $F_{inert,M}$ is converted into an inert gas flow at standard conditions, $F_{inert,M}^0$, to allow the calculation of J_{inert} via Eq. (S3.1). Conversion with $F_{inert,M}^0 = F_{inert,M} \cdot p_{feed} \cdot T^0 / (p^0 \cdot T_M)$ yields:

$$F_{inert,M}^0 = \frac{p_{feed} \cdot T^0}{p^0 \cdot T_M} \cdot A_M \cdot \left(\frac{2 \cdot (p_{feed} - p_{perm})}{\rho_{feed} \cdot \zeta_M^*}\right)^{0.5} \tag{S3.4}$$

To calculate $F_{inert,M}^0$ depending on the permeate pressure, p_{perm}, the pressure loss coefficient ζ_M^* is required, which was determined experimentally using the constant volume/variable pressure method.

Equipment for measuring the inert gas flow depending on the pressure gradient

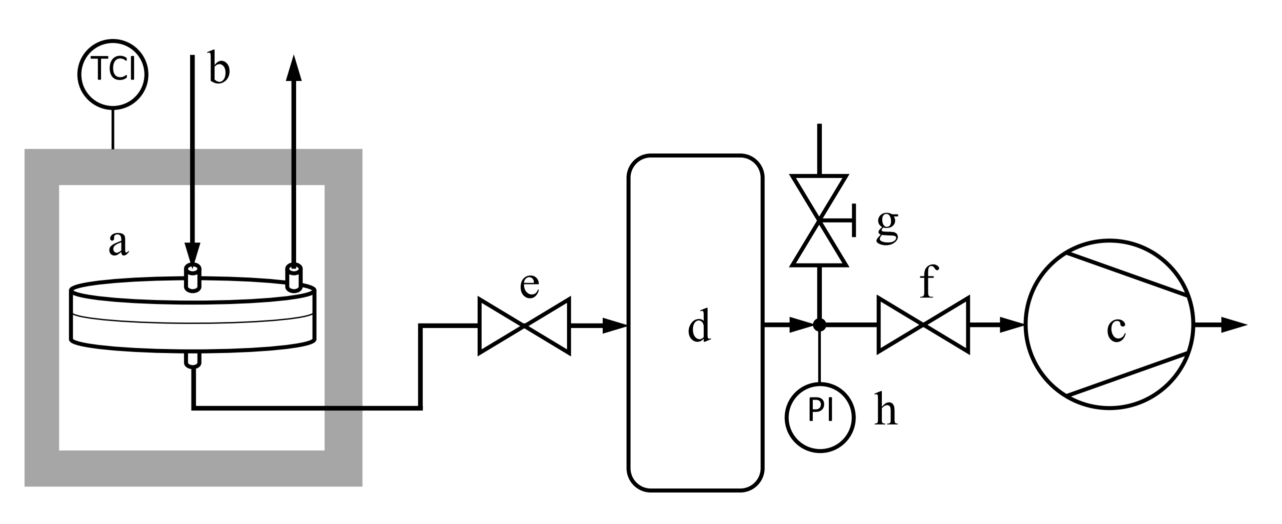

Figure S3.1 Equipment for measuring the inert gas flow through the membrane depending on the permeate pressure; the membrane module, operated at 40 °C in an insulation box (a), is permanently fed with nitrogen or air as an inert gas (b); the inert gas is transported through the membrane driven by a low permeate pressure ($p_{perm} < p_A$); the low permeate pressure is generated with a vacuum pump (c); a buffer volume of ca. 2.4 L (d) and two valves (e, f) are placed between the membrane module and the vacuum pump; another regulation valve (g) allows to adjust the permeate pressure to a specific value; the current permeate pressure is read on a pressure gauge (h); PVC tubes with fabric reinforcement were used for connecting all system components to each other; valve (e) allows to separate the apparatus from the membrane module; valve (f) is open and valve (g) is regulated when a defined permeate pressure is set, and both are closed when the inert gas flow is measured.

The inert gas flow through the membrane was measured using the constant volume/variable pressure method [Zhong et al. 2014, Yang et al. 2018, Macher et al. 2021, Shen et al. 2022]. The used equipment (Figure S3.1) is similar in design to the apparatus applied for the separation of ethyl acetate from artificial exhaust gas (Figure 1A in the main text). The key differences are as follows: the upper

side of the membrane is flushed with inert gas (nitrogen or air) without ethyl acetate, two additional valves are installed whereby valve (e) is used to separate the membrane from the apparatus and valve (f) is used to separate the vacuum pump from the apparatus.

The constant gas volume in the above-mentioned measuring method is the volume of the permeate gas being located between the membrane and valve (f). The main contribution to this gas volume comes from the buffer vessel with a capacity of approx. 2.4 liters (a vacuum-prove desiccator).

Characterization of the equipment for measuring $F^0_{inert,M}$

The equipment for measuring the inert gas flow is not completely leak-proof, so that some gas from the environment enters the evacuated apparatus, even if valves (e), (f) and (g) are closed, causing the pressure in the apparatus slowly to rise. This slow pressure increase interferes with the measurement of the pressure increase resulting from the inert gas flow through the membrane and must be taken into account by calculation.

The leakage flow depends on the pressure inside the apparatus (= p_{perm}) and the ambient pressure p_A. The higher the difference $p_A - p_{perm}$ is, the higher the leakage flow becomes. The leakage flow was therefore measured for various permeate pressures by using the already mentioned constant volume/variable pressure method. For this purpose, the apparatus was evacuated by the vacuum pump (valve (f) open, valve (e) and (g) closed). When the pressure inside the apparatus had fallen slightly below the target pressure, valve (f) was closed and the vacuum pump switched of. Then, a desired pressure was adjusted by slowly opening regulation valve (g) so that air could flow in and the pressure increased (observed by pressure gauge (h)). Valve (g) was closed when reaching the desired pressure. Then, the time-dependent increase of the pressure was observed resulting from the incoming ambient air (leakage flow).

The recorded $p_{perm}(t)$ data are used to describe the leakage flow ($F_{leakage}$) depending on p_{perm}. The following equation is valid:

$$\frac{dp_{perm}}{dt} = \frac{p_A}{V_{perm}} \cdot F_{leakage} \tag{S3.5}$$

Herein, p_A is the ambient pressure and V_{perm} is the gas volume at the permeate side between the valves (e) and (f). The temporal change of the permeate pressure was measured for several p_{perm} values (p_{perm} = 10, 200, 400, 600 or 800 mbar). The observed temporal changes $\Delta p_{perm}/\Delta t$ depending on p_{perm} are shown in Figure S3.2A and were converted into leakage flows by Eq. (S3.6) which was obtained from Eq. (S3.5):

$$F_{leakage} = \frac{V_{perm}}{p_A} \cdot \frac{\Delta p_{perm}}{\Delta t} \tag{S3.6}$$

The calculated leakage flows depending on p_{perm} are shown in Figure S3.2B. It becomes apparent that the $F_{leakage}$-p_{perm} dependency is non-linear.

A model equation was formulated for describing the observed $F_{leakage}$-p_{perm} dependency. Preliminary data analyses have shown that the leakage flow is caused by two different phenomena: by pore flow (leaks due to narrow gaps) and by diffusion (permeation of gas through the homogeneous tube

material). The pore flow is described with a term similar to Eq. (S3.3), and the flow through homogeneous material is formulated as gas diffusion with the 1st Fick's law. Both contributions to the total leakage flow are combined by addition:

$$F_{leakage} = k_1 \cdot \left(p_A - p_{perm}\right)^{0.5} + k_2 \cdot \left(p_A - p_{perm}\right) \quad \text{(S3.7)}$$

The parameters k_1 and k_2 summarize unknown variables. The model equation (S3.7) was then fitted to the measured $F_{leakage}$-p_{perm} data by varying both model parameters and searching for a combination of k_1 and k_2 that gives a minimum deviation between the measured and calculated data. An optimum adaptation of the model to the measured data were obtained for k_1 = 6.23·10^{-9} m^3 s^{-1} bar$^{-0.5}$ and k_2 = 5.20·10^{-9} m^3 s^{-1} bar^{-1} (see Figure S3.2B). The leakage model describes the measured data very well. The determined model parameters only apply to the specifically characterized test system; a modification in design would change parameters k_1 and k_2.

The observed leakage flow shown in Figure S3.1 increased with a decreasing permeate pressure. However, even the lowest permeate pressure of 10 mbar resulted in a leakage flow of no more than 40.4 mL h^{-1}. This value is quite small, but too large to be ignored.

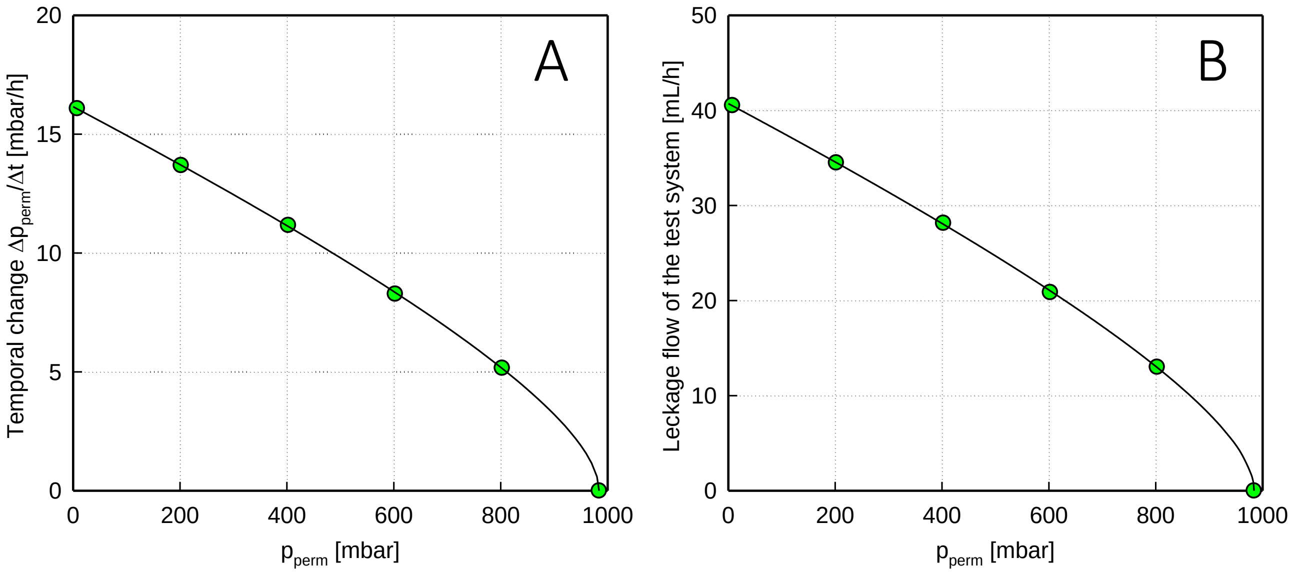

Figure S3.2 (A) Measured temporal changes $\Delta p_{perm}/\Delta t$ and (B) leakage gas flow, $F_{leakage}$, of the test system shown in Figure S3.1, both variables depending on the permeate pressure; determination by the constant volume/variable pressure method; symbols are measured values while solid lines represent model calculations; conditions and parameters: V_{perm} = 2.480 L, p_A = 984 mbar, T_A = 296.6 K; model calculations done with k_1 = 6.23·10^{-9} m^3 s^{-1} bar$^{-0.5}$, k_2 = 5.20·10^{-9} m^3 s^{-1} bar^{-1}.

Measurement of $F^0_{inert,M}$

According to Eq. (S3.4), the inert gas flow through the membrane $F^0_{inert,M}$ depends on the permeate pressure p_{perm} and is determined by the pressure loss coefficient ζ^*_M which is a characteristic parameter of the membrane. In the following, determination of parameter ζ^*_M by the constant volume/ variable pressure is demonstrated.

The change in pressure inside the test system is a result of the superimposition of the inert gas flow through the membrane and the leakage gas flow from the surroundings into the apparatus:

$$\frac{dp_{perm}}{dt} = \frac{p_A}{V_{perm}} \cdot (F_{inert,M} + F_{leakage}) \tag{S3.8}$$

Determination of parameter ζ_M^* requires information on the $F_{inert,M}$-p_{perm} dependency. For exploring the needed data, the test system shown in Figure S3.1 was used. The membrane module was fed with a flow of nitrogen (99.999 % N_2), the valves (e) and (f) were opened, the valve (g) was closed and the vacuum pump (c) was switched on to reduce the permeate pressure. When the pressure inside the test system reached 6 mbar, valve (f) was closed and the pump was switched off. The two gas flows $F_{inert,M}$ and $F_{leakage}$, driven by the vacuum inside the system, cause a temporal increase of pressure p_{perm} which was observed at the pressure gauge (h).

Such a measured $p_{perm}(t)$ dependency is shown in Figure S3.3A. The measured $p_{perm}(t)$ function was not linear since the relation between the gas flows $F_{inert,M}$ and $F_{leakage}$ and the pressure difference ($p_{feed} - p_{perm}$ = $p_A - p_{perm}$) is non-linear as it can be seen from Eqs. (S3.4) and (S3.7).

Eq. (S3.8) was used to calculate $F_{inert,M}$ depending on p_{perm} based on the measured $p_{perm}(t)$ data. The equation was converted to $F_{inert,M}$, the differential quotient was replaced by a difference quotient, and $F_{leakage}$ was substituted by Eq. (S3.7):

$$F_{inert,M} = \frac{V_{perm}}{p_A} \cdot \frac{\Delta p_{perm}}{\Delta t} - \left[k_1 \cdot \left(p_A - p_{perm}\right)^{0.5} + k_2 \cdot \left(p_A - p_{perm}\right)\right] \tag{S3.9}$$

The calculation was done based on the measured $p_{perm}(t)$ data shown in Figure S3.3A for pressure intervals of Δp_{perm} = 20 mbar and for the parameters listed in the legend of Figure S3.3. The calculated inert gas flow through the membrane $F_{inert,M}$ steadily decreases with time (Figure S3.3B) due to the temporally diminishing pressure gradient.

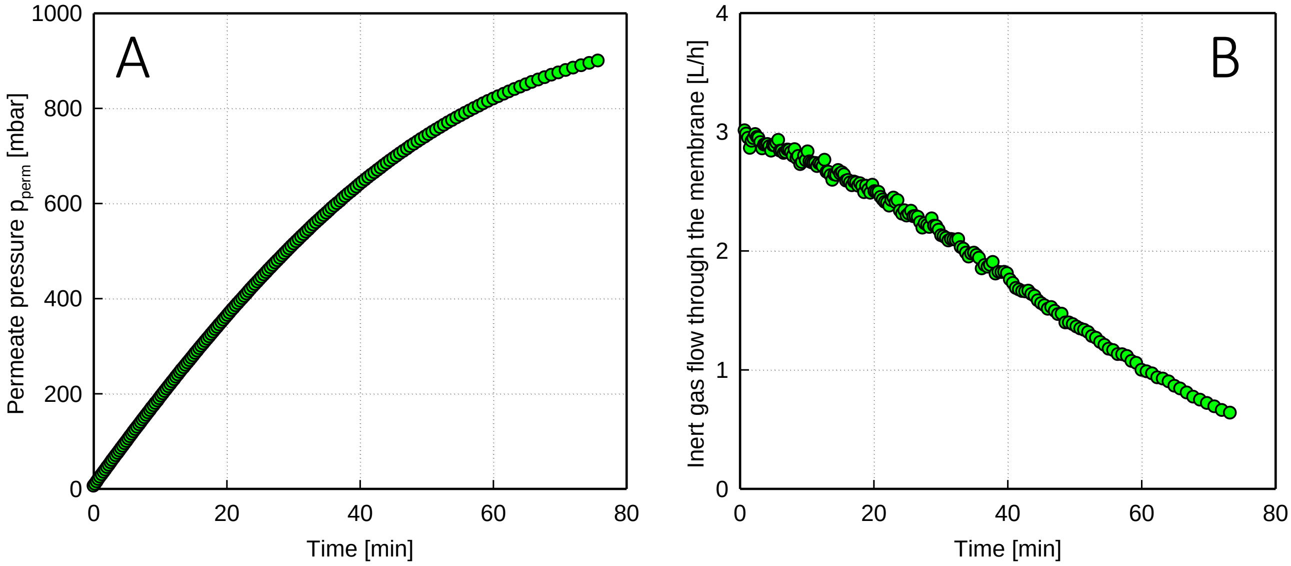

Figure S3.3 (A) Measured pressure p_{perm} and (B) inert gas flow through the tested membrane, $F_{inert,M}$, both depending on time; determination by the constant volume/variable pressure method; $F_{inert,M}$ calculated from the measured $p_{perm}(t)$ data by using Eq. (S3.9); feed gas consisting of 60 L h^{-1} N_2; conditions and parameters: V_{perm} = 2.568 L, $p_A = p_{feed}$ = 987 mbar, T_M = 313.1 K, T_A = 298.1 K, ρ_{feed} = 1.062 kg m^{-3}; leakage flow calculated with $k_1 = 6.23 \cdot 10^{-9}$ m^3 s^{-1} $bar^{-0.5}$ and $k_2 = 5.20 \cdot 10^{-9}$ m^3 s^{-1} bar^{-1}.

Next, the inert gas flows through the membrane, $F_{inert,M}(t)$, were converted to gas flows under standard conditions $F_{inert,M}^{0}(t)$ (at p^0 = 1013.25 mbar and T^0 = 273.15 K) and presented as a function of the permeate pressure $F_{inert,M}^{0}(p_{perm})$. Then, Eq. (S3.4) was fitted to these $F_{inert,M}^{0}(p_{perm})$ data by varying the unknown pressure loss coefficient ζ_M^* (see Figure S3.4C).

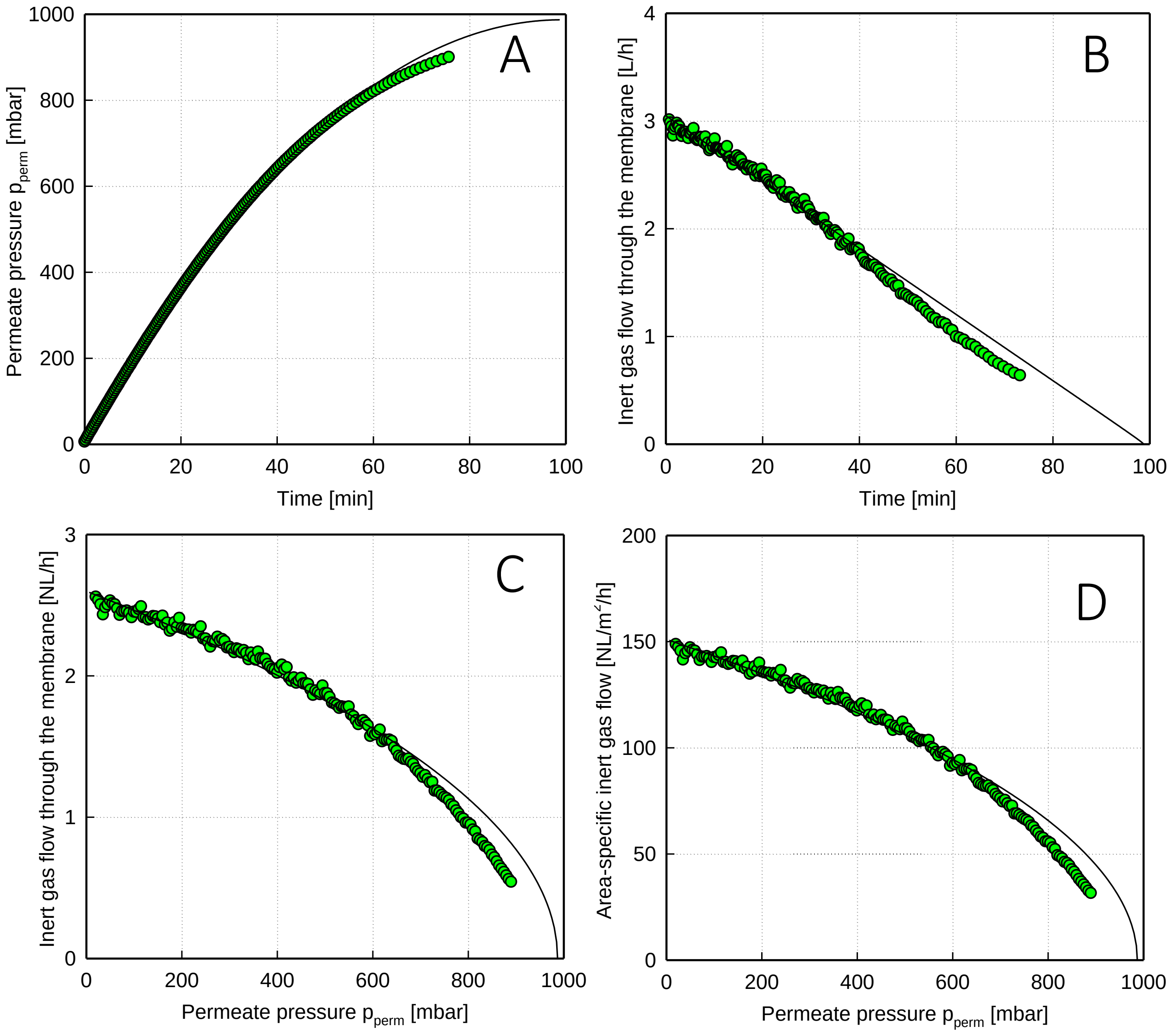

Figure S3.4 (A) Permeate pressure p_{perm} and (B) inert gas flow through the tested membrane $F_{inert,M}$, both depending on time, (C) inert gas flow $F_{inert,M}^{0}$ and (D) the area-specific inert gas flow $F_{inert,M}^{0}/A_M$, both given under standard conditions and depending on the permeate pressure p_{perm}; experimental data taken from Figure S3.3; solid lines represent model calculations; feed gas consisting of 60 L h^{-1} N_2; conditions and parameters: V_{perm} = 2.568 L, $p_A = p_{feed}$ = 987 mbar, T_M = 313.1 K, T_A = 298.1 K, ρ_{feed} = 1.062 kg m^{-3}, A_M = 0.0172 m^2; model calculations done with k_1 = 6.23·10^{-9} m^3 s^{-1} bar$^{-0.5}$, k_2 = 5.20·10^{-9} m^3 s^{-1} bar^{-1}, and ζ_M^* = 7.62·10^{13}.

In case of the here presented data, an optimum adaptation of the model to the measured data were obtained for a pressure loss coefficient of ζ_M^* = 7.62·10^{13} (see Figure S3.4). The deviations between the measured values and the model calculations are small for p_{perm} < 600 mbar. But for p_{perm} > 600 mbar, deviations become larger; however, this is not relevant because all separation experiments were done for p_{perm} = 10 ... 500 mbar. The leakage gas flow was distinctly smaller than the inert gas flow ($F_{leakage}/F_{inert,M} \approx 0.01$; compare Figure S3.3B with Figure S3.2B).

During the experiments on separation of ethyl acetate with a membrane module, the pressure loss coefficient ζ_M^* was repeatedly determined with the constant volume/variable pressure method. Over a separation period of 150 h, the pressure loss coefficient increased at first rapidly, then more and more slowly, and lastly approached asymptotically a constant value of ζ_M^* = 6·10^{14}. The increasing pressure loss coefficient correlated with a decreasing inert gas flow; within the separation period of 150 h, the inert gas flow reduced by the factor of three. This effect of a temporally decreasing inert gas flow has also been described in the literature [Baker et Low 2014, Ismail et al. 2015]. All separation experiments published in the main text were carried out after the inert gas flow as well as the pressure loss coefficient had reached constant values.

Comparison of measured $F_{inert,M}^0$ with calculated $F_{inert,M}^0$ values

The permeate gas flow cannot be routinely measured during separation experiments due to the low permeate pressure. The permeate gas flow is therefore calculated by balancing the membrane separation process (see Chapter 3.1 in the main part of this work), which resulted in Eq. (8) derived there. This equation allows to calculate F_{perm}^0 depending on known parameters (F_{feed}^0, $x_{EA,feed}$, $x_{H_2O,feed}$, $F_{inert,M}^0$) and measured variables ($x_{EA,ret}$, $x_{H_2O,ret}$). Actually, the parameters $x_{EA,feed}$, $x_{H_2O,feed}$ and $F_{inert,M}^0$ were also measured in advance ($x_{EA,feed}$ and $x_{H_2O,feed}$ by mass spectrometry and $F_{inert,M}^0$ with the constant volume/variable pressure method as explained above).

To check the reliability of Eq. (8), the permeate gas flow was measured in separation experiments and compared with calculated F_{perm}^0 values. Two different methods were used to determine the permeate gas flow: (1) F_{perm}^0 was measured by the constant volume/variable pressure method as described above, and (2) F_{perm}^0 was obtained from $F_{perm}^0 = F_{feed}^0 - F_{ret}^0$, whereby F_{ret}^0 was measured using a bubble flow meter.

The higher the feed gas flow with ethyl acetate was, the larger the permeate gas flow became (Figure S3.5). This observation can be explained by the fact that F_{feed}^0 takes influence on the flux of ethyl acetate through the membrane. A rising value of F_{feed}^0 increases the concentration of ethyl acetate at the upper side of the membrane which heightens the gradient of the ester across the membrane and thus increases the permeation of ethyl acetate through it (see the main part of this work). The permeate gas flow F_{perm}^0 is the sum of $F_{inert,M}^0$ and the ester flow $F_{EA,M}^0$. The inert gas flow $F_{inert,M}^0$ is constant throughout the measurements (p_{perm} is always 10 mbar, and the ester does not influence $F_{inert,M}^0$), but the increase of F_{feed}^0 enlarges $F_{EA,M}^0$ which changes F_{perm}^0 (= $F_{inert,M}^0 + F_{EA,M}^0$).

The calculated F_{perm}^0 curve describes the measured data quite well (Figure S3.5). In case of F_{feed}^0 = 0, no ethyl acetate is fed to the membrane module so that only inert gas passes through the membrane, which is why the curve starts at F_{perm}^0 = $F_{inert,M}^0$. The increasing flow F_{feed}^0 let the calculated F_{perm}^0 increase as well. At very large F_{feed}^0 values, F_{perm}^0 asymptotically approaches a maximum value (not shown). The deviations between the measured and calculated permeate flows become larger at larger feed gas flows due to the susceptibility to errors of the measurement methods at high gas flows. This especially applies to the measurement of F_{ret}^0 with the bubble flow meter.

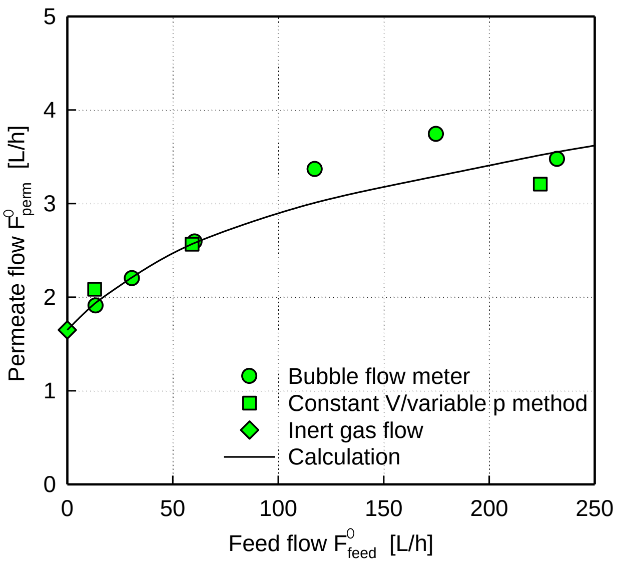

Figure S3.5 Permeate gas flow F^0_{perm} through the membrane depending on the feed gas flow F^0_{feed} at a defined content of ethyl acetate in the feed gas and a specific permeate pressure; measurement of F^0_{perm} by the volume/variable pressure method and indirectly as $F^0_{perm} = F^0_{feed} - F^0_{ret}$ with determination of F^0_{ret} using a bubble flow meter; calculation of F^0_{perm} by Eq. (8) of the main part of this work; symbols represent permeate flows measured with the two methods, while the solid line is the calculated permeate gas flow; the artificial feed gas was based on air with a water content of 0.013 L L^{-1} and an ester content of $x_{EA,feed}$ = 0.025 L L^{-1}; conditions and parameters: p_{perm} = 10 mbar, p_A = p_{feed} = 995.5 mbar, T_M = 313.1 K; the inert gas flow was determined to be $F^0_{inert,M}$ = 1.65 L h^{-1} (ρ_{feed} = 1.107 kg m^{-3}, A_M = 0.0172 m^2, ζ^*_M = 1.84·10^{14}).

Possible influence of ethyl acetate on $F^0_{inert,M}$

The literature reports that the polymer matrix of mixed-matrix membranes can swell due to absorption of the transported compound, causing the microscopic defects in the membrane to narrow and the inert gas flow to become smaller [Yu et al. 2011, Suleman et al. 2016].

In order to check whether this effect also occurs with the composite membrane used in this work, the apparatus shown in Figure 1A was applied. The feed gas of the membrane module consisted of air with 0.013 L L^{-1} water and a varied content of ethyl acetate. In these measurements, the permeate gas consisted of a mixture of inert gas and ethyl acetate. The total gas flow through the membrane (i.e., the sum of $F^0_{inert,M}$ and $F^0_{EA,M}$) was measured using the constant volume/variable pressure method.

The data in Figure S3.6 clearly show that the permeate flow is increased by the presence of ethyl acetate in the feed gas since ethyl acetate passes through the membrane together with the inert gas. The more ethyl acetate the feed gas contained, the larger the flow of ethyl acetate was and the greater the total gas flow through the membrane became. The gas flow through the membrane caused the permeate pressure gradually to increase and the permeate flow continuously to decrease since the permeate pressure as the driving force for the ester transport through the membrane steadily diminished. At higher permeate pressures, the F_{perm}-p_{perm} graphs approached each other, because then hardly any ethyl acetate was transported through the membrane (see the Y_{EA}-p_{perm} dependency in Figure 2 in the main part of this work) and the permeate gas mainly consisted of inert gas.

In addition to the measured data, Figure S3.6 also shows the permeate gas flow depending on p_{perm} for the case that the feed gas does not contain any ethyl acetate; this curve corresponds to the inert gas flow through the membrane and was calculated with Eq. (S3.4) using the currently valid pressure

loss coefficient ζ_M^*. The measured F_{perm}-p_{perm} data approach this calculated curve asymptotically at higher permeate pressures; the then measured permeate flows correspond to the inert gas flow. This fact proves that $F_{inert,M}^0$ is not influenced by the presence of ethyl acetate in the feed gas.

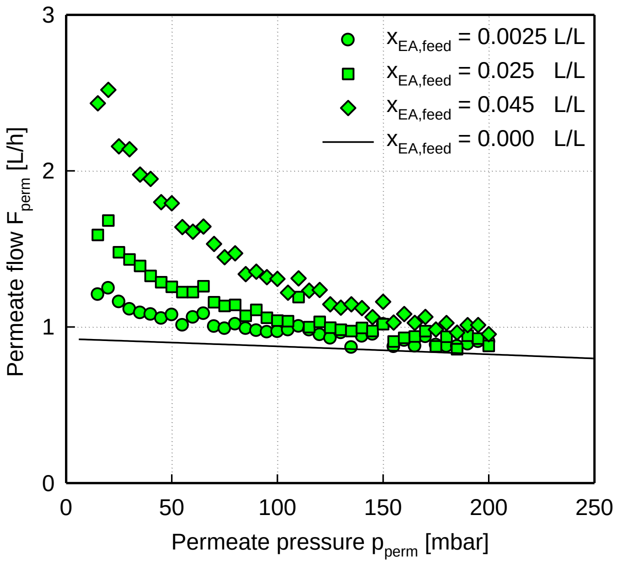

Figure S3.6 Permeate gas flow through the membrane depending on the permeate pressure p_{perm} and on the content of ethyl acetate in the feed gas as indicated in the figure legend; symbols represent permeate flows measured with the constant volume/variable pressure method, while the solid line is the inert gas flow calculated with Eq. (S3.4); the feed gas was based on air with a water content of 0.013 L L^{-1}; conditions and parameters: F_{feed}^0 = 60 L h^{-1}, V_{perm} = 2.568 L, $p_A = p_{feed}$ = 985.5 mbar, T_M = 313.1 K; the calculation of $F_{inert,M}^0$ was done with the given parameters and ρ_{feed} = 1.096 kg m^{-3}, A_M = 0.0172 m^2, and $\zeta_M^* = 6 \cdot 10^{14}$.

Literature

Baker, R.W., Low, B.T., Gas separation membrane materials: a perspective. *Macromol.* 2014, *47*, 6999–7013.

Ismail, A.F., Khulbe, K.C., Matsuura, T., *Gas separation membranes*, Springer Int. Publishing, Basel 2015.

Macher, J., Hausberger, A., Macher, A.E., Morak, M., et al., Critical review of models for H_2-permeation through polymers with focus on the differential pressure method. *Int. J. Hydrog. Energy* 2021, *46*, 22574–22590.

Shen, B., Zhao, S., Yang, X., Carta, M., et al., Relation between permeate pressure and operational parameters in VOC/nitrogen separation by a PDMS composite membrane. *Sep. Purif. Technol.* 2022, *280*, 119974.

Suleman, M.S., Lau, K.K., Yeong Y.F., Plasticization and swelling in polymeric membranes in CO_2 removal from natural gas. *Chem. Eng. Technol.* 2016, *39*, 1604–1616.

Xu, G., Liang, F., Yang, Y., Hu, Y., et al., An improved CO_2 separation and purification system based on cryogenic separation and distillation theory. *Energy* 2014, *7*, 3484–3502.

Yang, W., Zhou, H., Zong, C., Li, Y., et al., Study on membrane performance in vapor permeation of VOC/N_2 mixtures via modified constant volume/variable pressure method. *Sep. Purif. Technol.* 2018, *200*, 273–283.

Yu, M., Noble, R.D., Falconer, J.L., Zeolite membranes: microstructure characterization and permeation mechanisms. *Acc. Chem. Res.* 2011, *44*, 1196–1206.

Zhong, W., Li, X., Liu, F., Tao, G., et al., Measurement and correlation of pressure drop characteristics for air flow through sintered metal porous media. *Transp. Porous Media* 2014, *101*, 53–67.
