# Supplementary material for: In situ Product Recovery of Microbially Synthesized Ethyl Acetate from the Exhaust Gas of a Bioreactor by Membrane Technology
Source: Eng Life Sci. 2024 Sep 30;24(12):e202400041. doi: 10.1002/elsc.202400041 (PMC11620624; doi:10.1002/elsc.202400041)
Supplement: Supplementary file 4 — Supplementary information [file ELSC-24-e202400041-s004.pdf]

Supporting Information 4:

Coupling of two and more membrane modules in series

In situ product recovery of microbially synthesized ethyl acetate from the exhaust gas of a bioreactor by membrane technology

Andreas Hoffmann, Alexander Franz, Christian Löser, Thomas Hoyer, Marcus Weyd, Thomas Walther

Used symbols are listed in the main part of the work or are explained here in the text.

These considerations focus on the separation yield of a membrane unit consisting of several membrane modules which are connected in series, as shown in Figure S4.1. The retentate gas leaving the first module is supplied as the feed gas to the second module and so on. The permeate flows of the single modules are combined to one summarized permeate flow, as depicted in Figure S4.1 for two modules. In these considerations it is assumed that the separation yields for ethyl acetate of the single membrane modules ($Y_{EA,1}$, $Y_{EA,2}$, ...) are known from balancing the separation process.

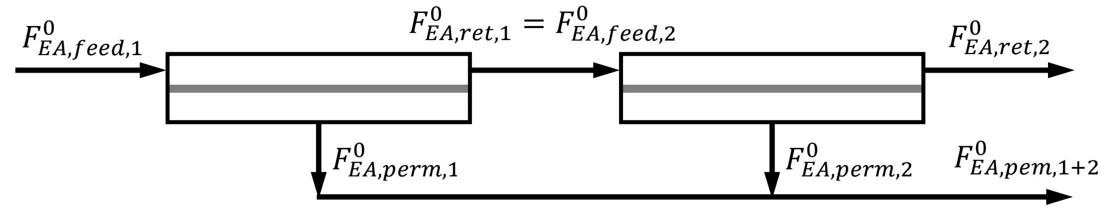

Figure S4.1 Membrane unit consisting of two identical membrane modules connected in series; flow rates of ethyl acetate given under standard conditions (p^0 = 1013.25 mbar, T^0 = 273.15 K); the volume fractions of ethyl acetate in the single flows exhibit the same notation ($x_{EA,feed,1}$, $x_{EA,ret,1}$ and so on).

Next, an equation is derived for calculating the separation yield of a membrane unit consisting of two membrane modules. Analogous to Eq. (2) in the main text of this work, the following applies to the separation yield of ethyl acetate of this membrane unit:

$$Y_{EA,1+2} = \frac{F^0_{EA,perm,1+2}}{F^0_{EA,feed,1}} = \frac{F^0_{EA,perm,1} + F^0_{EA,perm,2}}{F^0_{EA,feed,1}} \qquad \text{(S4.1)}$$

The numerator contains the flow of the separated ethyl acetate (identical to the sum of the two partial flows of both modules) and the denominator contains the total flow of ethyl acetate supplied to the membrane unit. Both permeate flows in the numerator are then replaced with $F^0_{EA,perm,i} = Y_{EA,i} \cdot F^0_{EA,feed,i}$:

$$Y_{EA,1+2} = \frac{Y_{EA,1} \cdot F^0_{EA,feed,1} + Y_{EA,2} \cdot F^0_{EA,feed,2}}{F^0_{EA,feed,1}} \qquad \text{(S4.2)}$$

For the flow of ethyl acetate fed to the second module, the following equation is true: $F^0_{EA,feed,2} = F^0_{EA,ret,1} = F^0_{EA,feed,1} - F^0_{EA,perm,1}$. Substitution of $F^0_{EA,feed,2}$ in equation (S4.2) using this relation results in:

$$Y_{EA,1+2} = \frac{Y_{EA,1} \cdot F^0_{EA,feed,1} + Y_{EA,2} \cdot (F^0_{EA,feed,1} - F^0_{EA,perm,1})}{F^0_{EA,feed,1}} \tag{S4.3}$$

Substituting $F^0_{EA,perm,1}$ again with $F^0_{EA,perm,1} = Y_{EA,1} \cdot F^0_{EA,feed,1}$ yields:

$$Y_{EA,1+2} = \frac{Y_{EA,1} \cdot F^0_{EA,feed,1} + Y_{EA,2} \cdot (F^0_{EA,feed,1} - Y_{EA,1} \cdot F^0_{EA,feed,1})}{F^0_{EA,feed,1}} \tag{S4.4}$$

Shortening gives $Y_{EA,1+2} = Y_{EA,1} + Y_{EA,2} \cdot (1 - Y_{EA,1})$, and further rearrangement finally results in:

$$Y_{EA,1+2} = 1 - (1 - Y_{EA,1}) \cdot (1 - Y_{EA,2}) \tag{S4.5}$$

If this consideration is extended to three or more membrane modules connected in series in the same manner as done for two modules, it can be shown that the following general relationship applies:

$$Y_{EA,1\ldots n} = 1 - \prod_{i=1}^{n} (1 - Y_{EA,i}) \tag{S4.6}$$

This equation is also valid for a single module (i.e., for n = 1). Assuming that all membrane modules connected in series achieve the same separation yield, the result is simplified (Y_{EA} is the separation yield of a single module):

$$Y_{EA,1\ldots n} = 1 - (1 - Y_{EA})^n \tag{S4.7}$$

Figure S4.2 shows the effect of different numbers of identical membrane modules connected in series on the separation yield of the entire membrane unit a varied separation yield of the single modules.

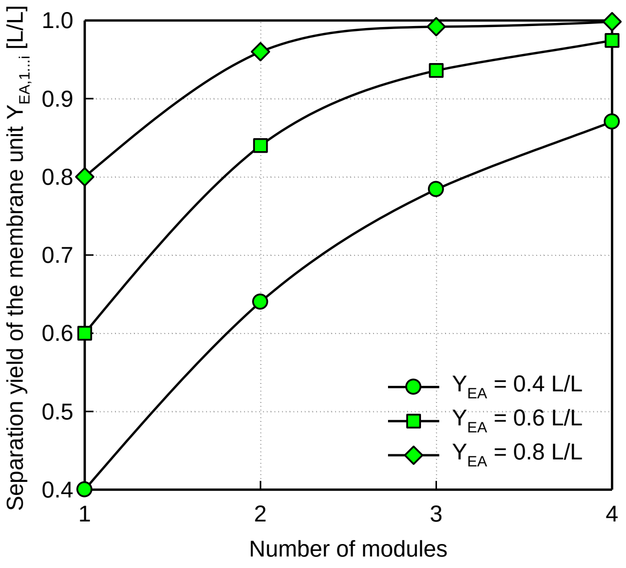

Figure S4.2 Separation yield of diverse membrane units consisting several numbers of identical membrane modules connected in series, assuming different separation yields of the single modules; calculation by Eq. S4.7

It becomes clearly visible from Figure S4.2 that the larger the number of modules in the membrane unit is, the higher the fraction of the separated ethyl acetate becomes. The separation yield $Y_{EA,1\dots n}$ approaches the value of 1 L L^{-1} (i.e., complete separation) when the number of connected membrane is high even if the separation yield of the single modules, Y_{EA}, would be low. When the separation yield of single modules is higher, e.g. Y_{EA} = 0.8 L L^{-1}, then $Y_{EA,1\dots n}$ becomes nearly 1 L L^{-1} at a number of three connected modules.

However, it should be noted that a larger number of membrane modules increases the design effort and therefore also the costs. In addition, the number of single modules correlates with the inert gas flow through the membrane, which dilutes the ethyl acetate in the permeate, making it more difficult to condense the separated ester.
