# Supplementary material for: In situ Product Recovery of Microbially Synthesized Ethyl Acetate from the Exhaust Gas of a Bioreactor by Membrane Technology
Source: Eng Life Sci. 2024 Sep 30;24(12):e202400041. doi: 10.1002/elsc.202400041 (PMC11620624; doi:10.1002/elsc.202400041)
Supplement: Supplementary file 5 — Supplementary information [file ELSC-24-e202400041-s002.pdf]

Supporting Information 5:

Water content of the gas flows during separation of ethyl acetate from real exhaust gas of a bioreactor

In situ product recovery of microbially synthesized ethyl acetate from the exhaust gas of a bioreactor by membrane technology

Andreas Hoffmann, Alexander Franz, Christian Löser, Thomas Hoyer, Marcus Weyd, Thomas Walther

Used symbols are listed in the main part of the work or are explained here in the text.

In this separation experiment, the exhaust gas of a bioreactor with microbial production of ethyl acetate was supplied to two identical membrane modules connected to each other in series (cultivation and separation conditions as given in the captures of Figures 4 and 5 in the main text). The composition of the feed and retentate gases of both membrane modules were analyzed by mass spectrometry. Balancing the separation process allowed to calculate the water content of the permeate gases.

The first module (Figure S5.1A) was fed with the exhaust gas of the bioreactor which was characterized by a constant water content of $x_{H_2O,feed,1}$ = 0.013 L L^{-1}. Part of the water passed through the membrane of the first module so that the water content of its retentate was reduced (look at $x_{H_2O,ret,1}$ in Figure S5.1A). The water content of $x_{H_2O,ret,1}$ equates to the water content of the feed of the second module ($x_{H_2O,feed,2}$ in Figure S5.1B). Some more water was separated in the second module so that $x_{H_2O,ret,2}$ was permanently lower than $x_{H_2O,feed,2}$.

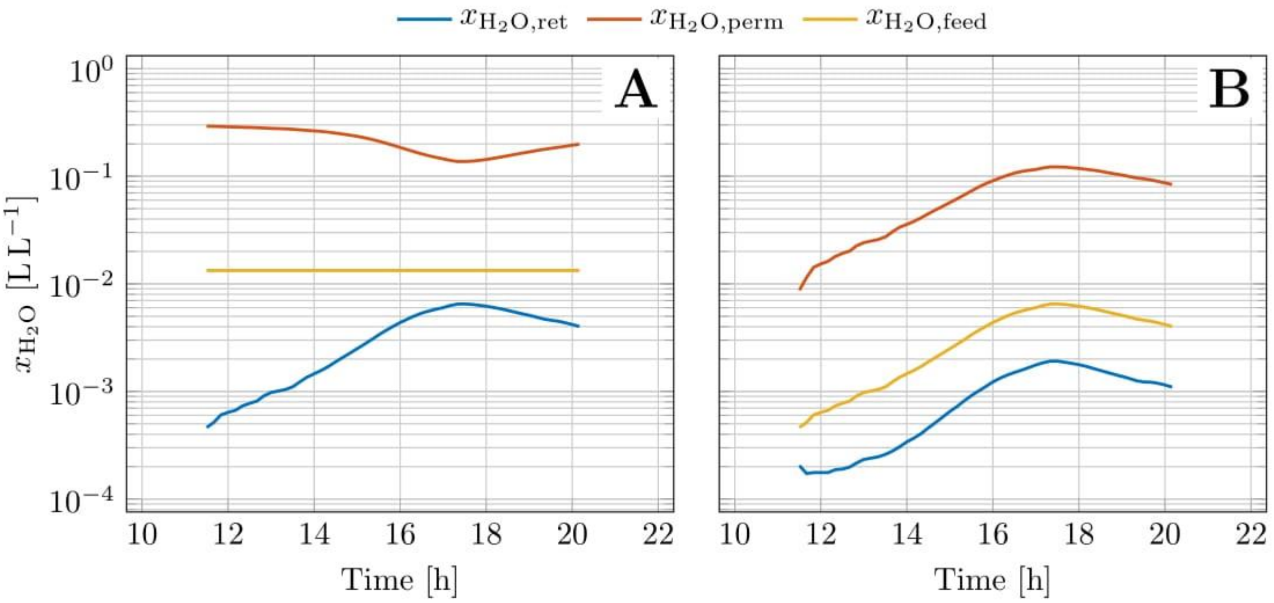

Figure S5.1 Water content in the feed gas, the retentate gas and the permeate gas of (A) the first module and (B) the second module of a membrane unit consisting two modules connected to each other in series; the $x_{H_2O,feed}$ and $x_{H_2O,ret}$ values were measured by mass spectrometry while $x_{H_2O,perm}$ values were calculated by balancing the separation process; the feed gas of the first module was the exhaust gas of a bioreactor (process shown in Figure 4 of the main text) from which microbially formed and stripped ethyl acetate was separated (look at Figure 5 of the main text)

Interestingly, the water permeation through the membrane of the first module distinctly depended on the process time. In the early process stage of separation, a high fraction of water passed through the membrane so that $x_{H_2O,ret,1}$ was much lower than $x_{H_2O,feed,1}$, and $x_{H_2O,perm,1}$ was quite high. As the process progressed, $x_{H_2O,ret,1}$ successively increased and $x_{H_2O,perm,1}$ decreased. The maximum $x_{H_2O,ret,1}$ and minimum $x_{H_2O,perm,1}$ values corresponded with the highest $x_{EA,feed,1}$ and largest $J_{EA,1}$ values. The flux of water through the membrane of the first module is obviously influenced by the ester content of the feed gas and or the flux of ethyl acetate through the membrane.

In the case of the second module, the flux of water through the membrane was much lower than in the first module (Figure S5.2) which can be explained by the reduced content of water in the feed gas $x_{H_2O,feed,2}$ compared to $x_{H_2O,feed,1}$ (Figure S5.1), due to the partial separation of water in the first module. Therefore, the flow of water through the second membrane, $F_{H_2O,perm,2}$, was largest when $x_{H_2O,feed,2}$ was highest.

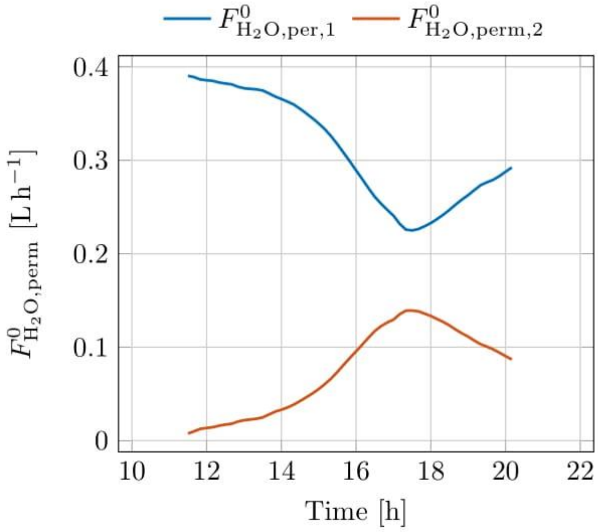

Figure S5.2 Flow of water through the membranes of two membrane modules connected to each other in series; the $F_{H_2O,perm}$ values were calculated by balancing the separation process; the feed gas of the first module was the exhaust gas of a bioreactor (process shown in Figure 4 of the main text) from which microbially formed and stripped ethyl acetate was separated (look at Figure 5 of the main text)
